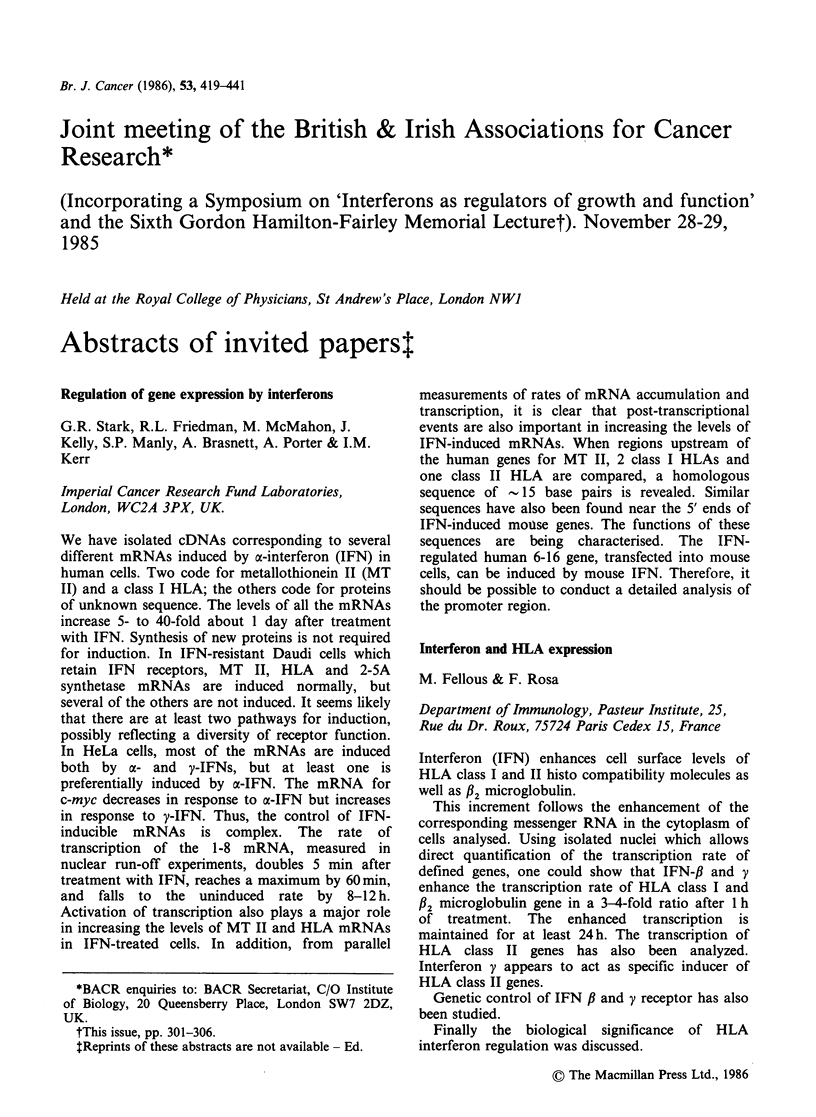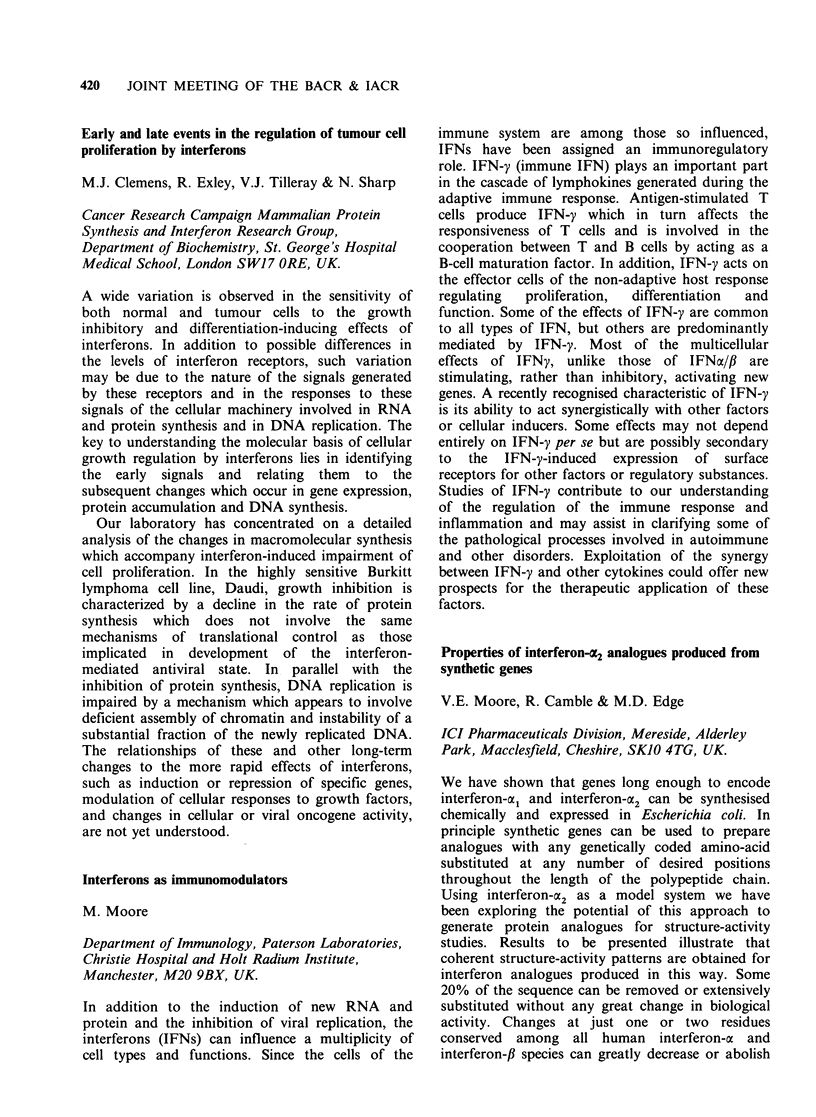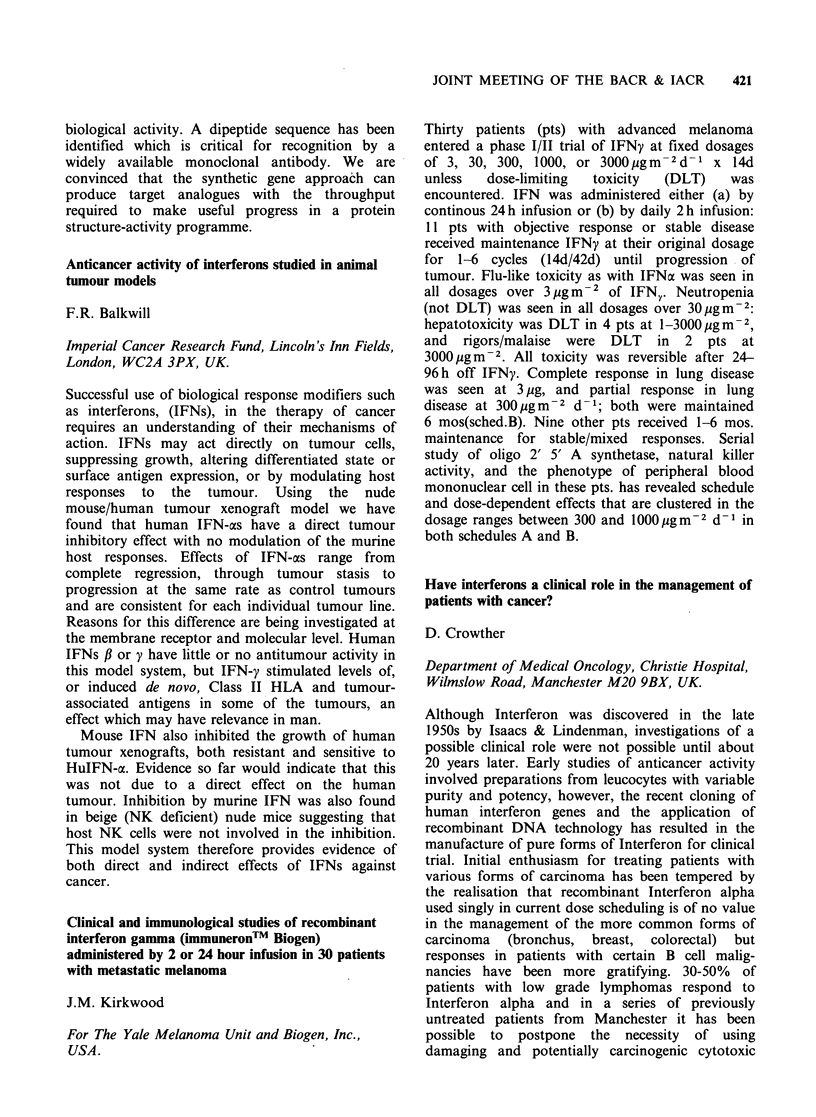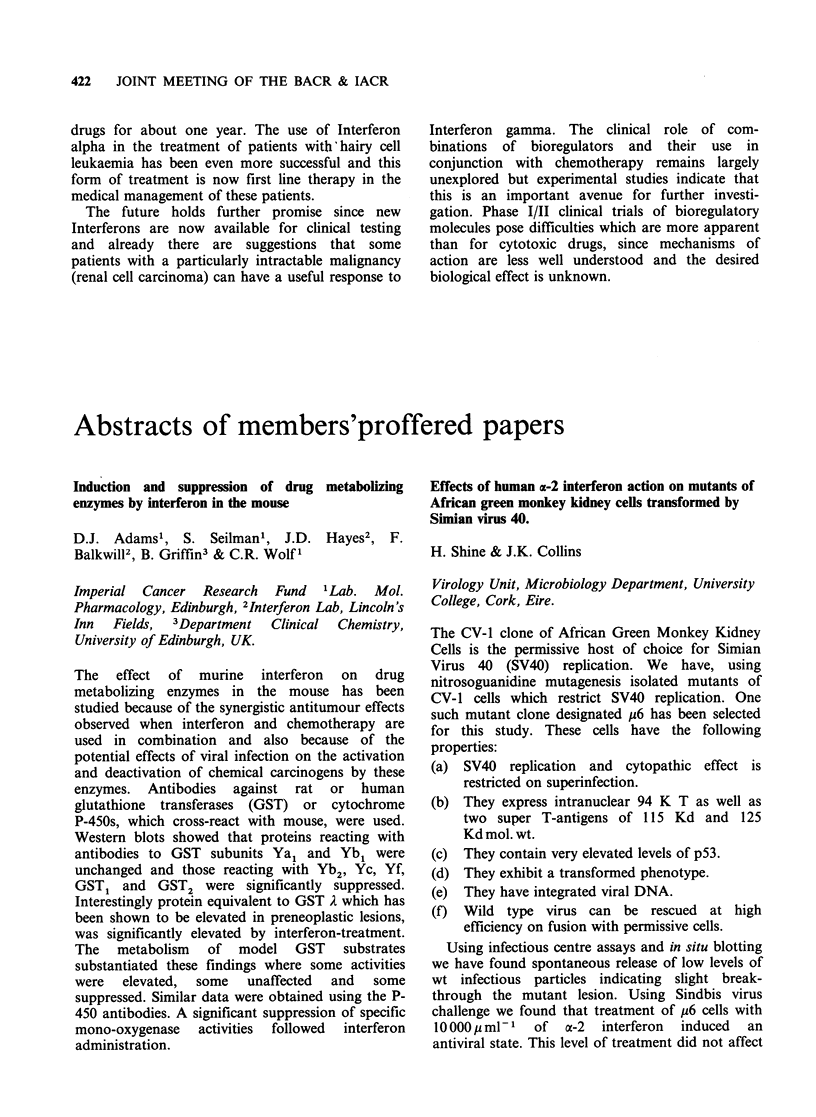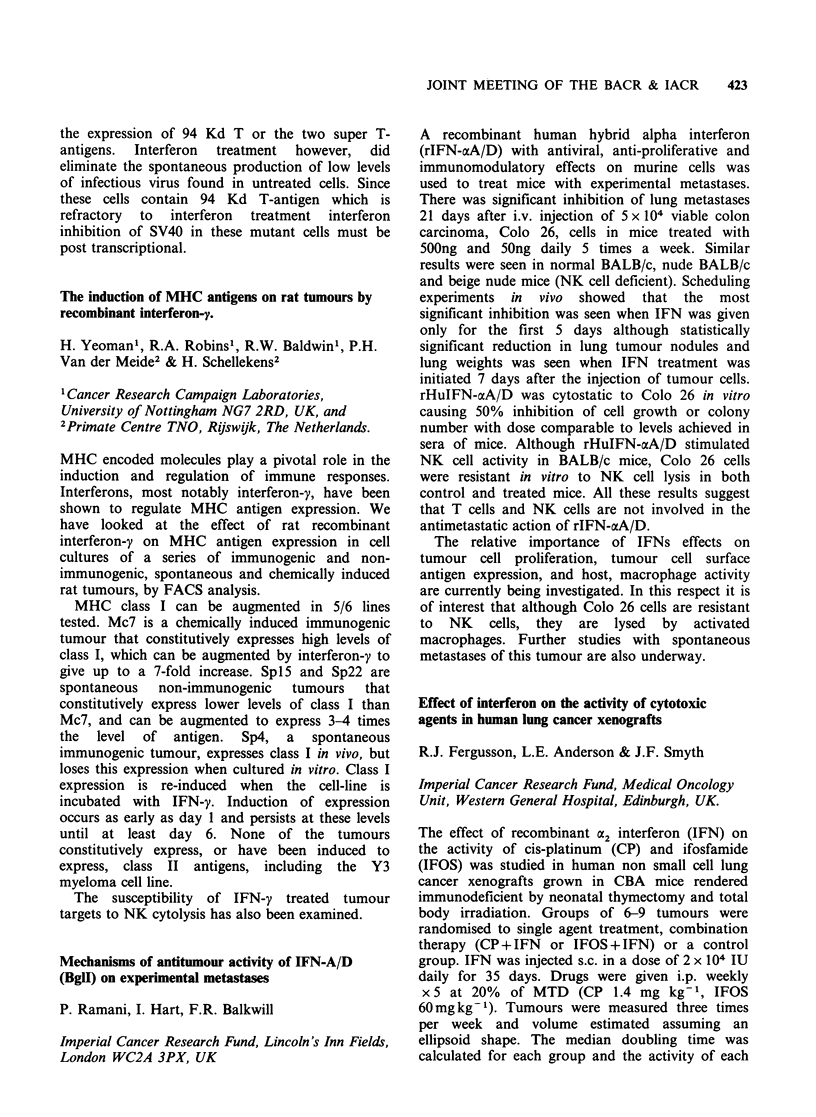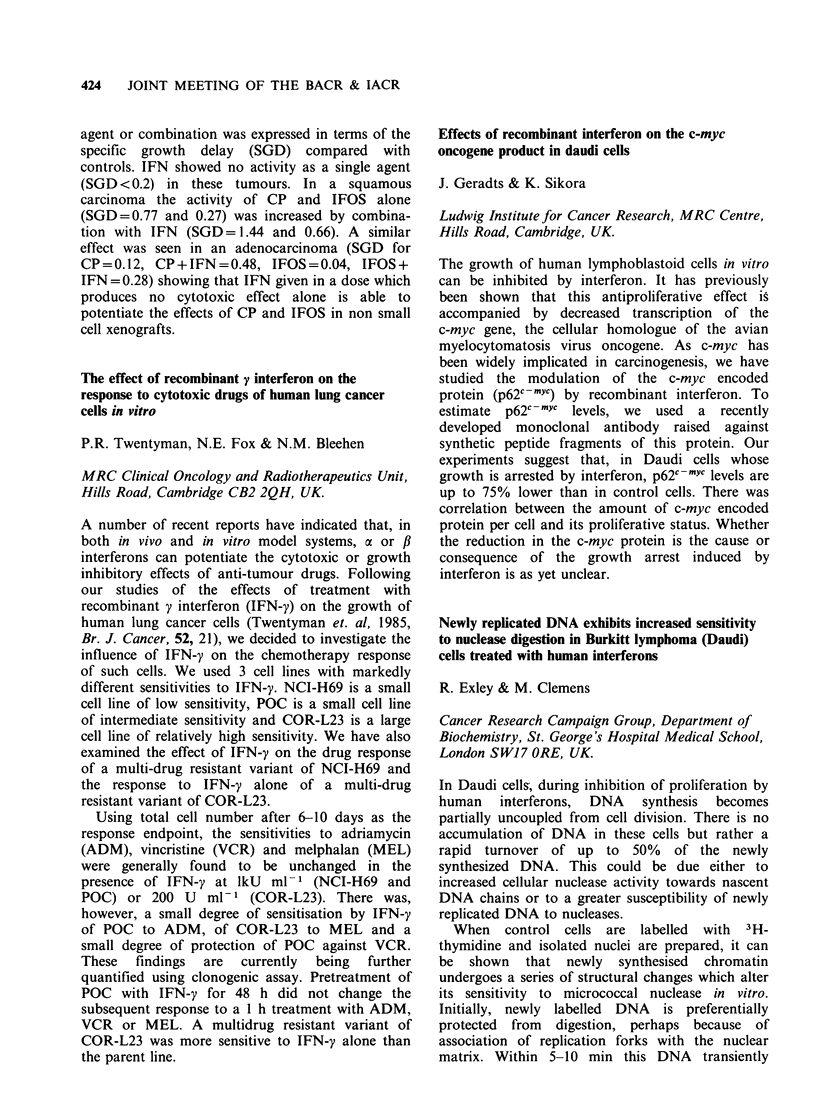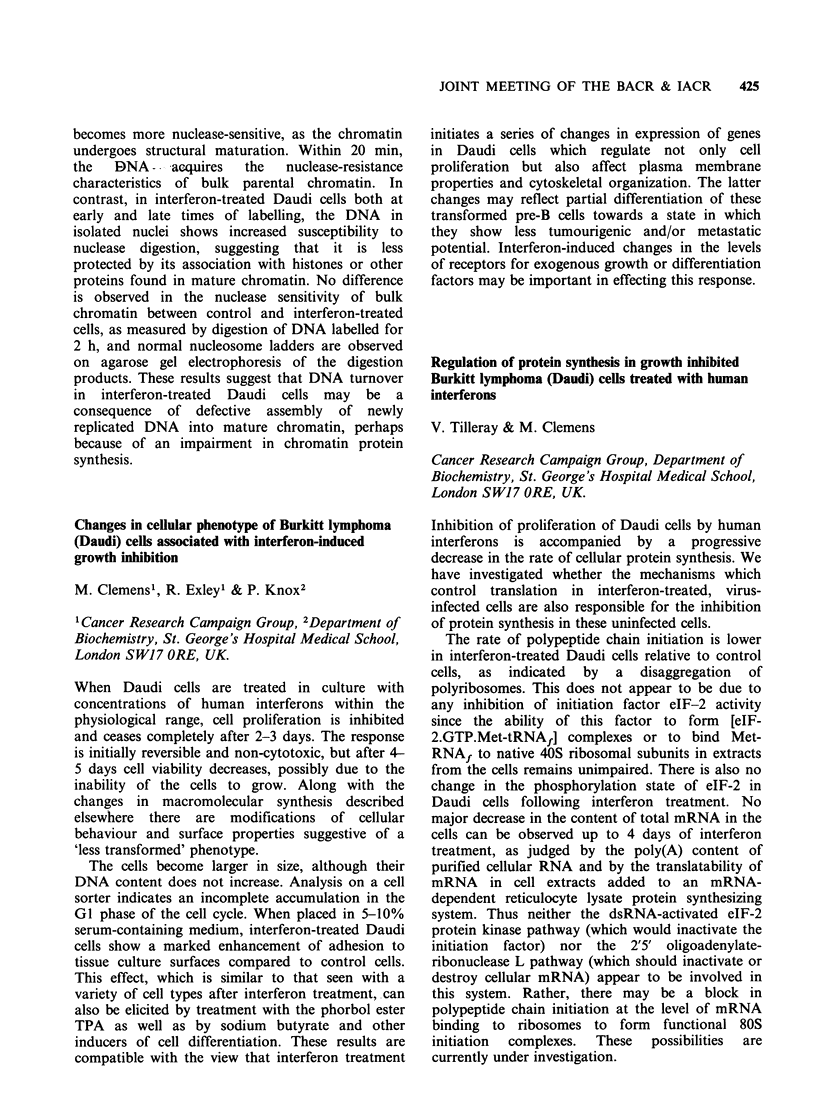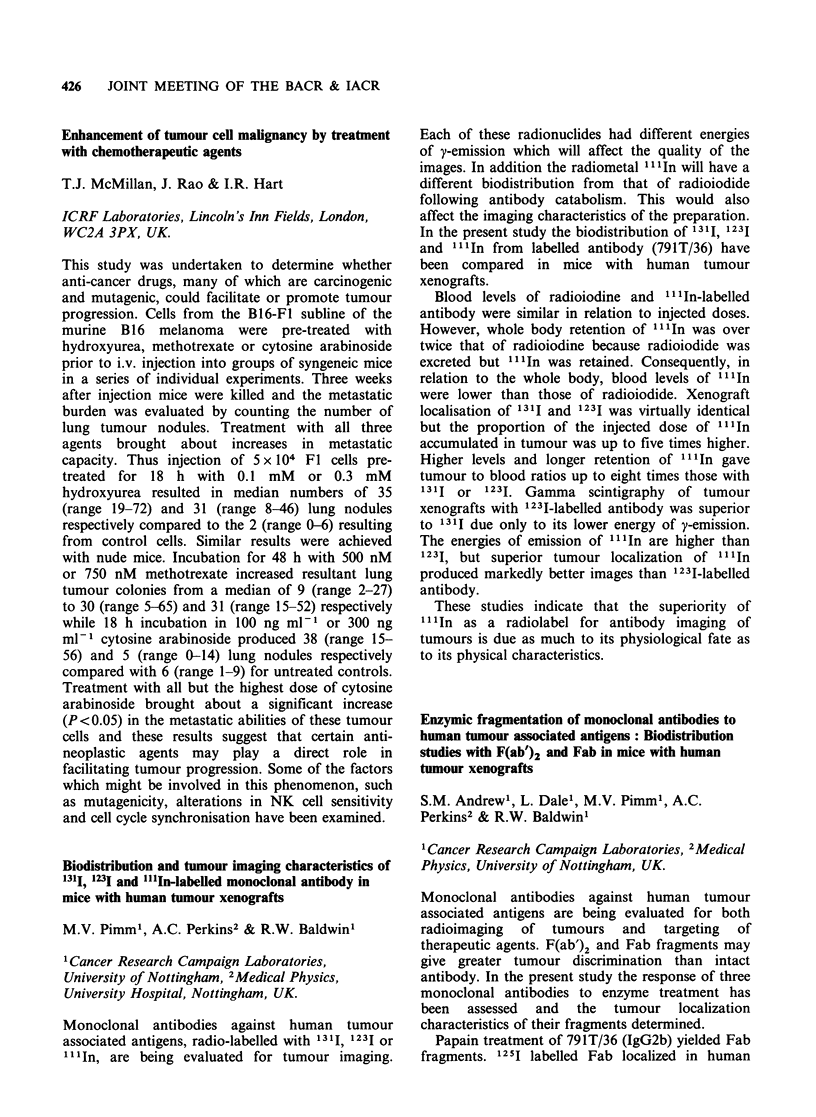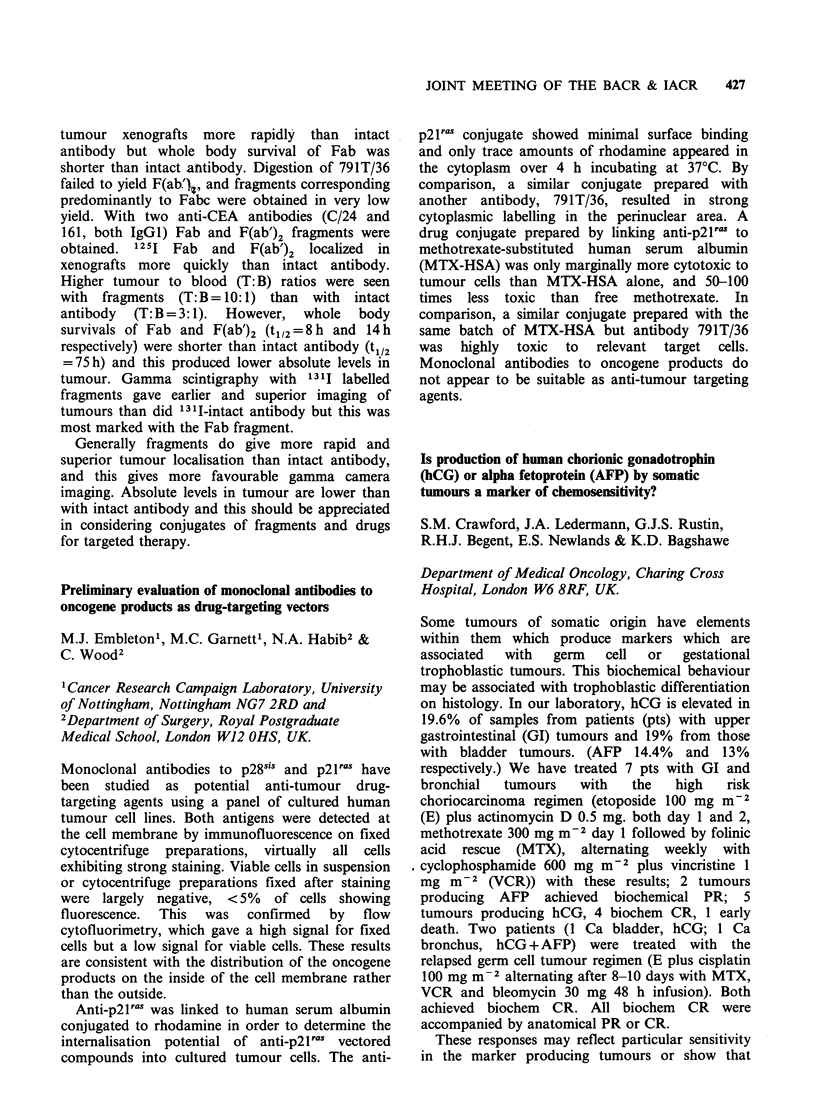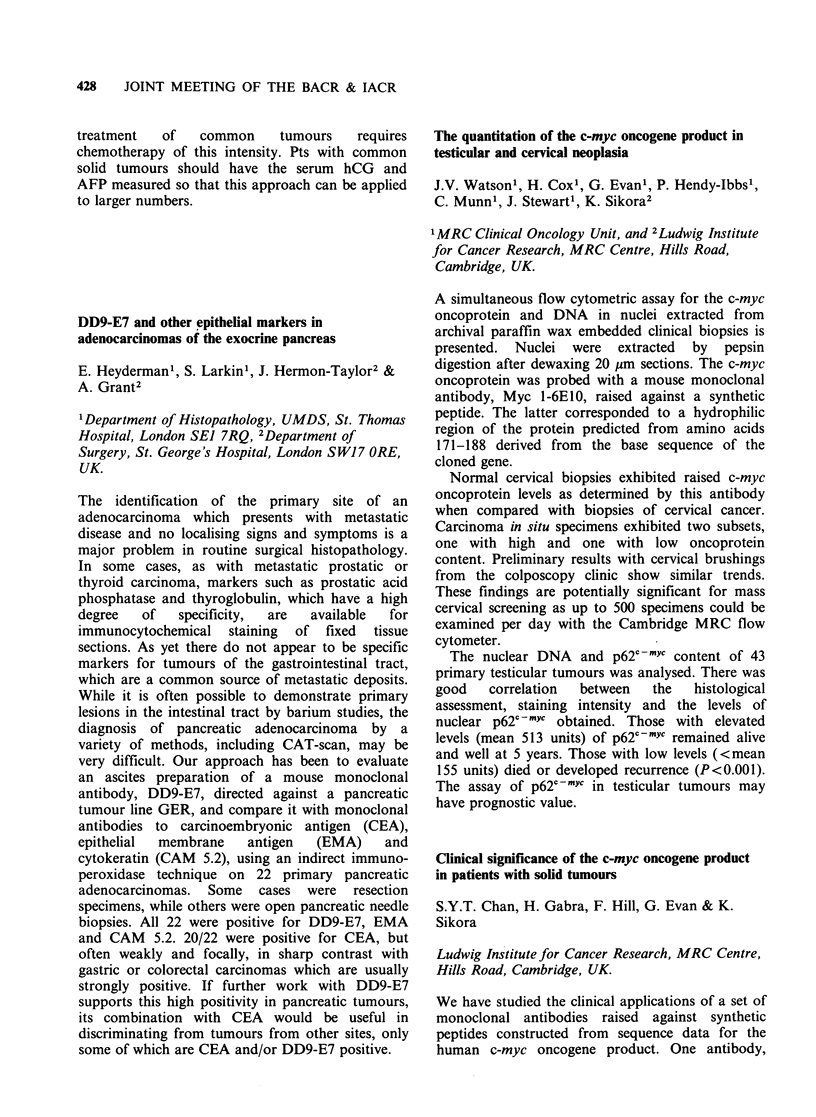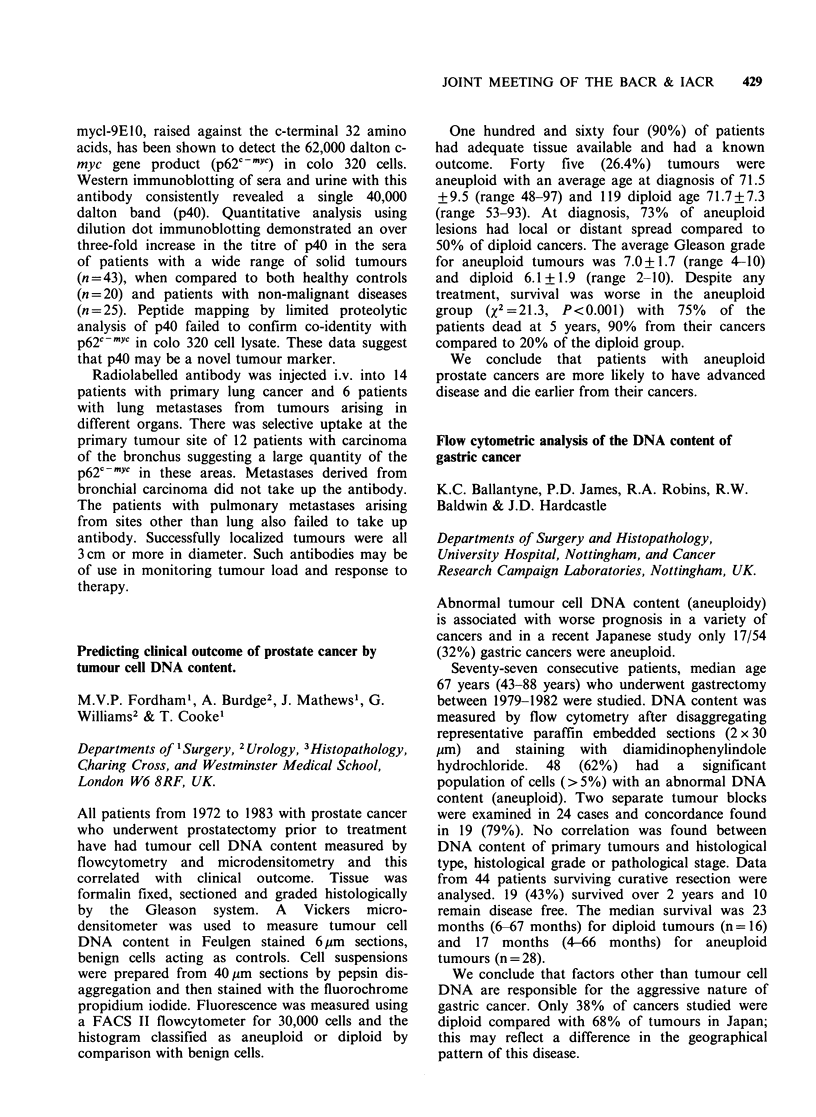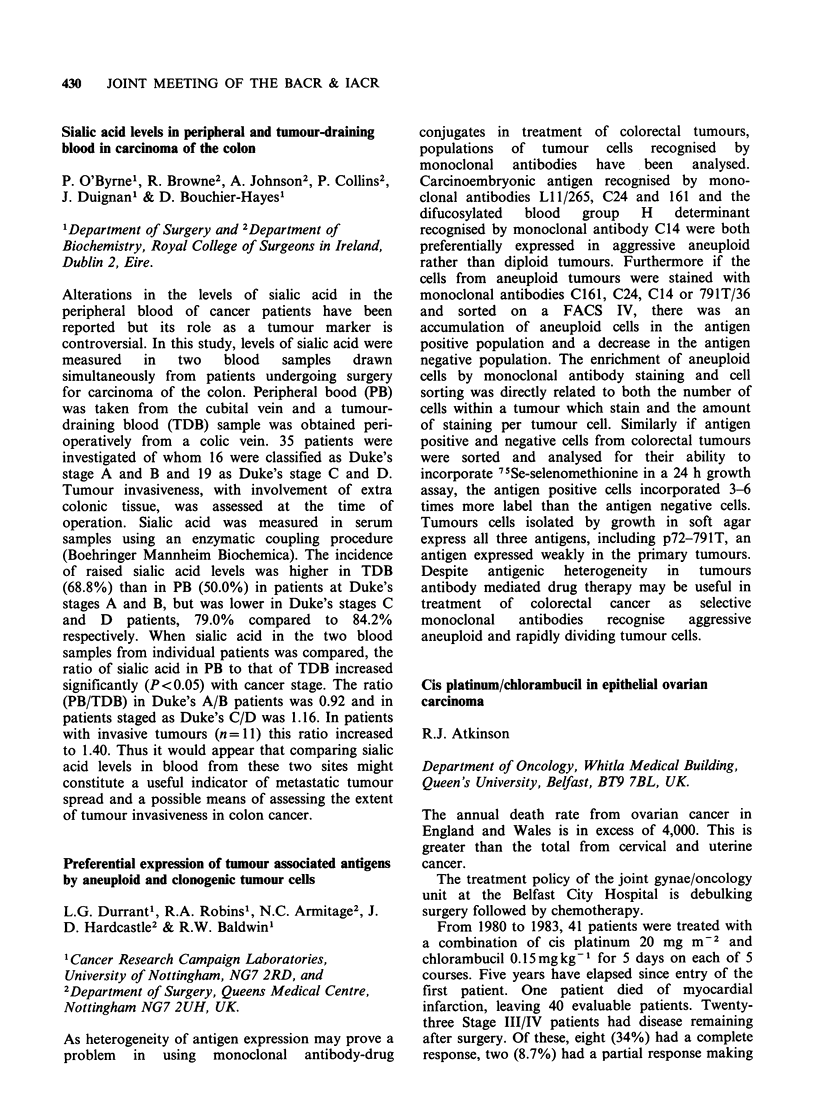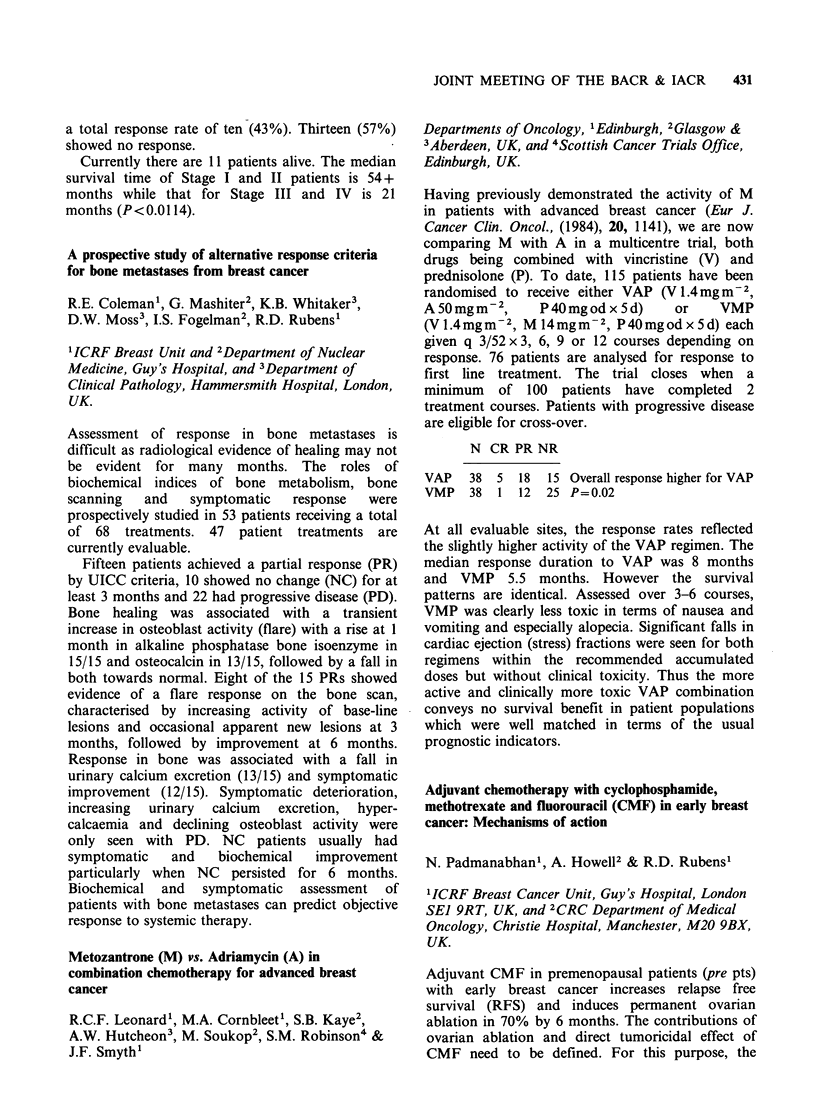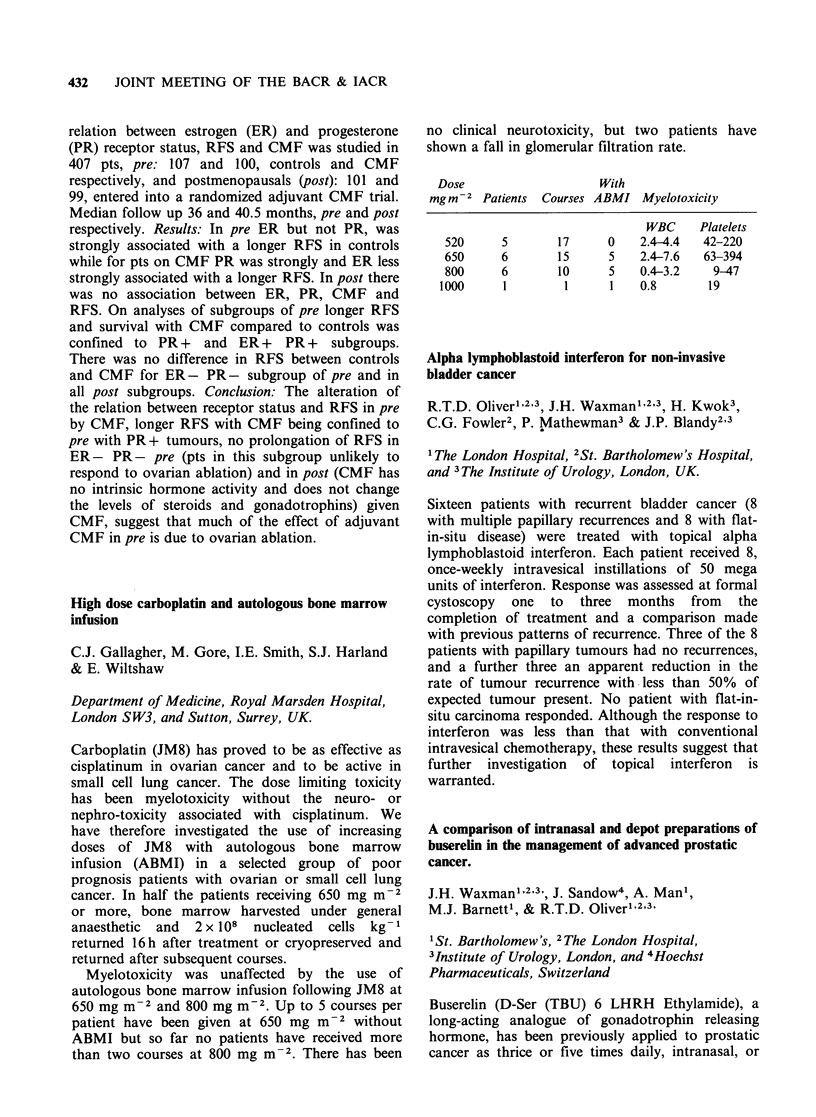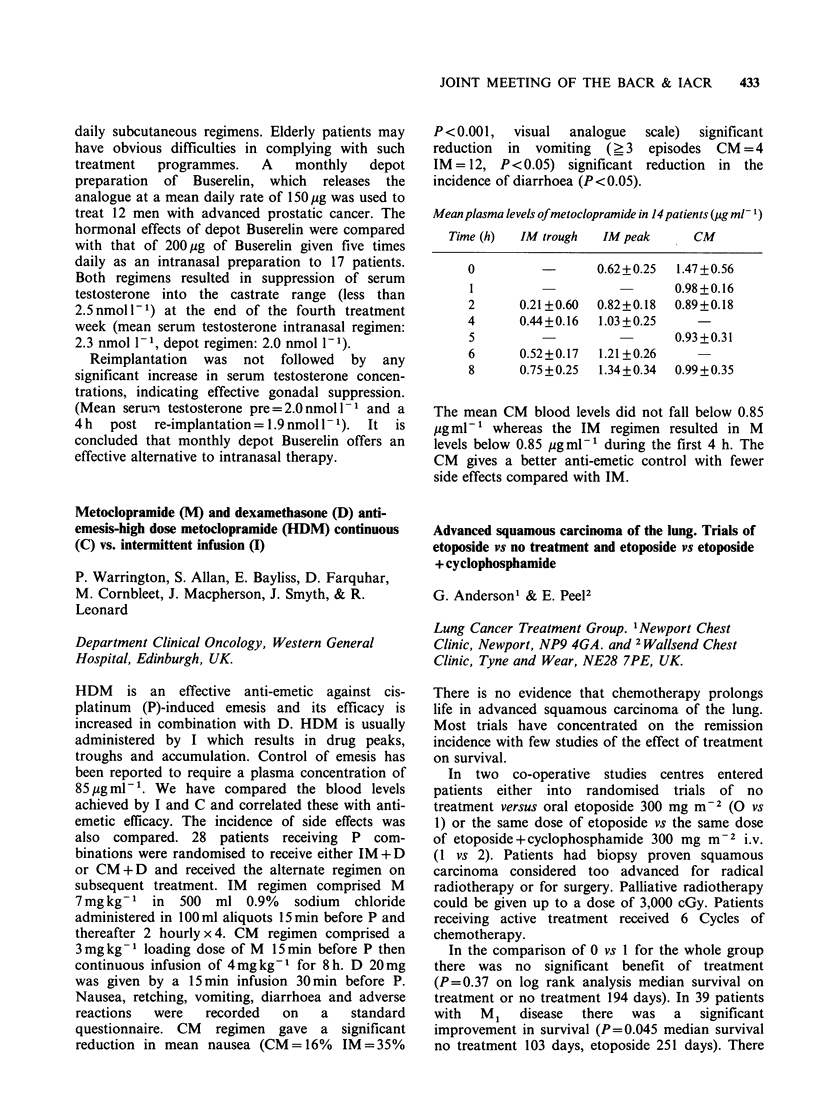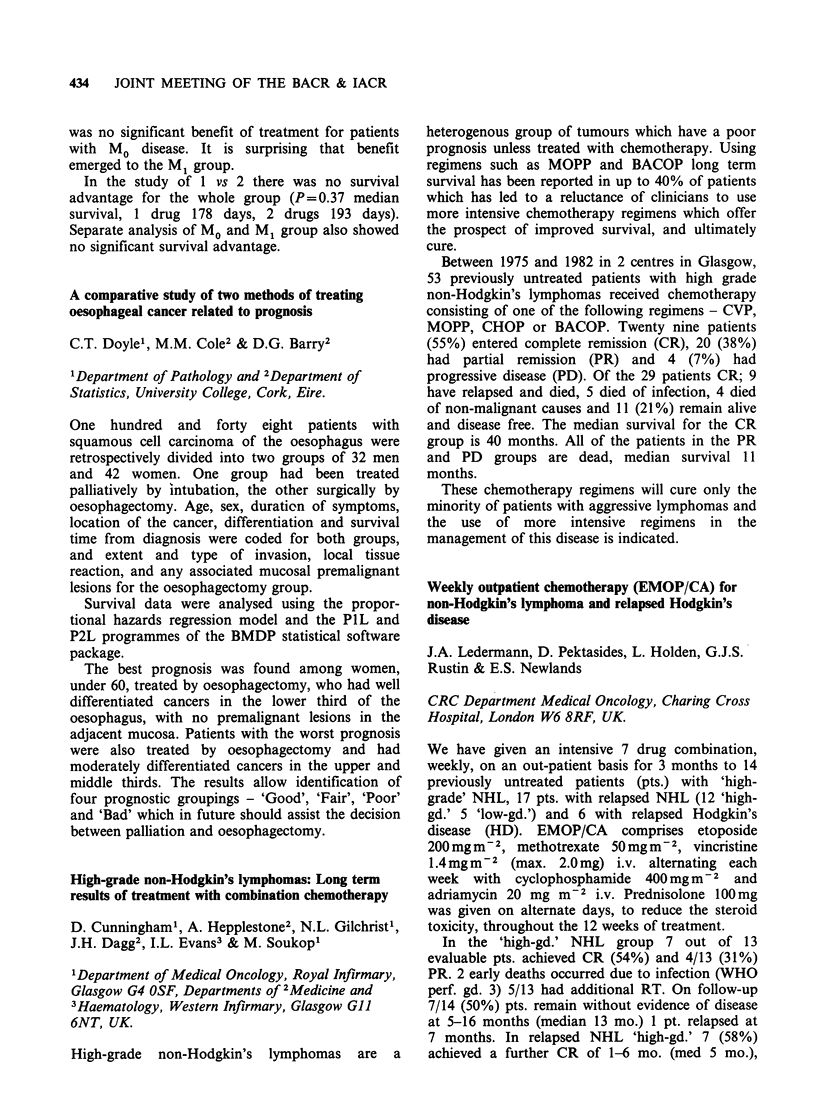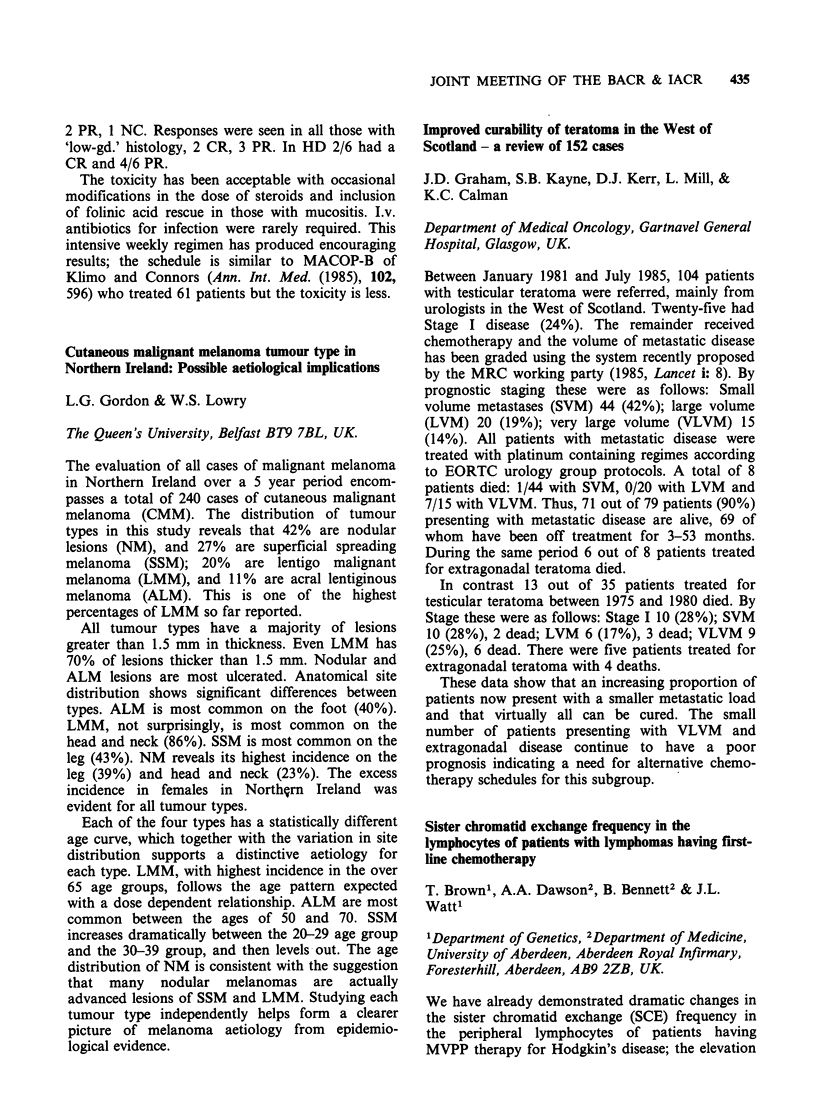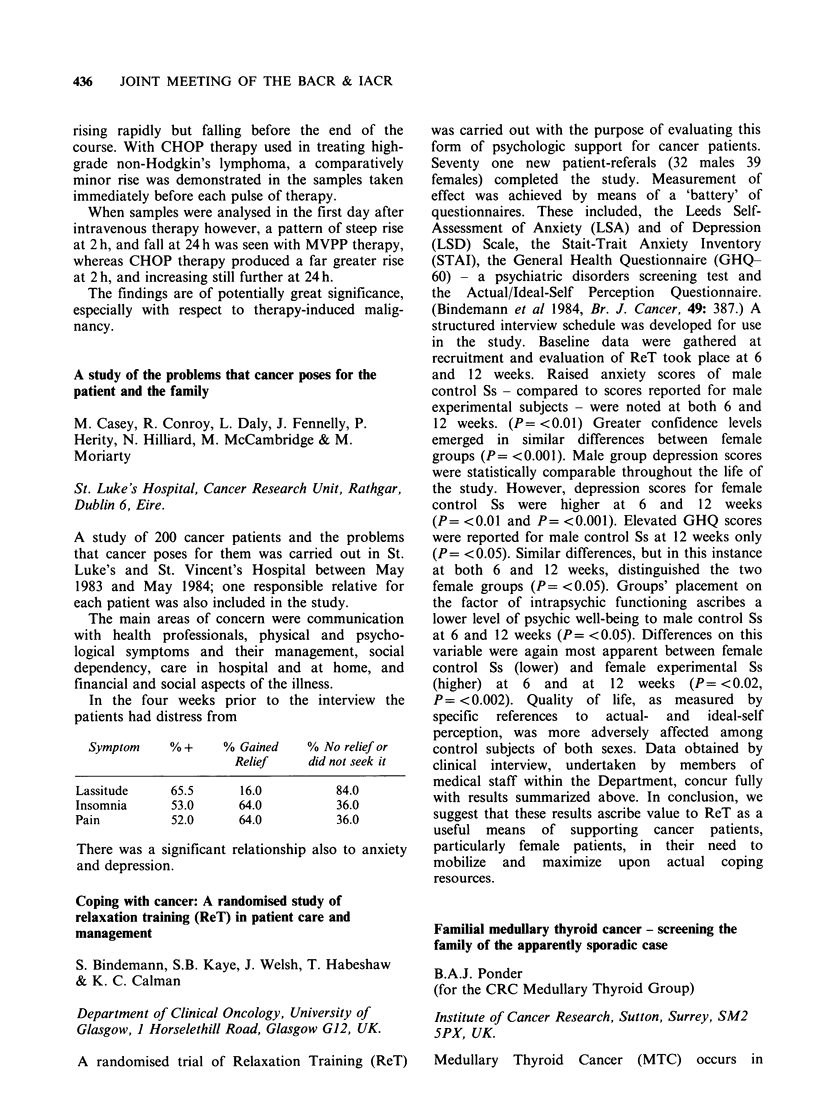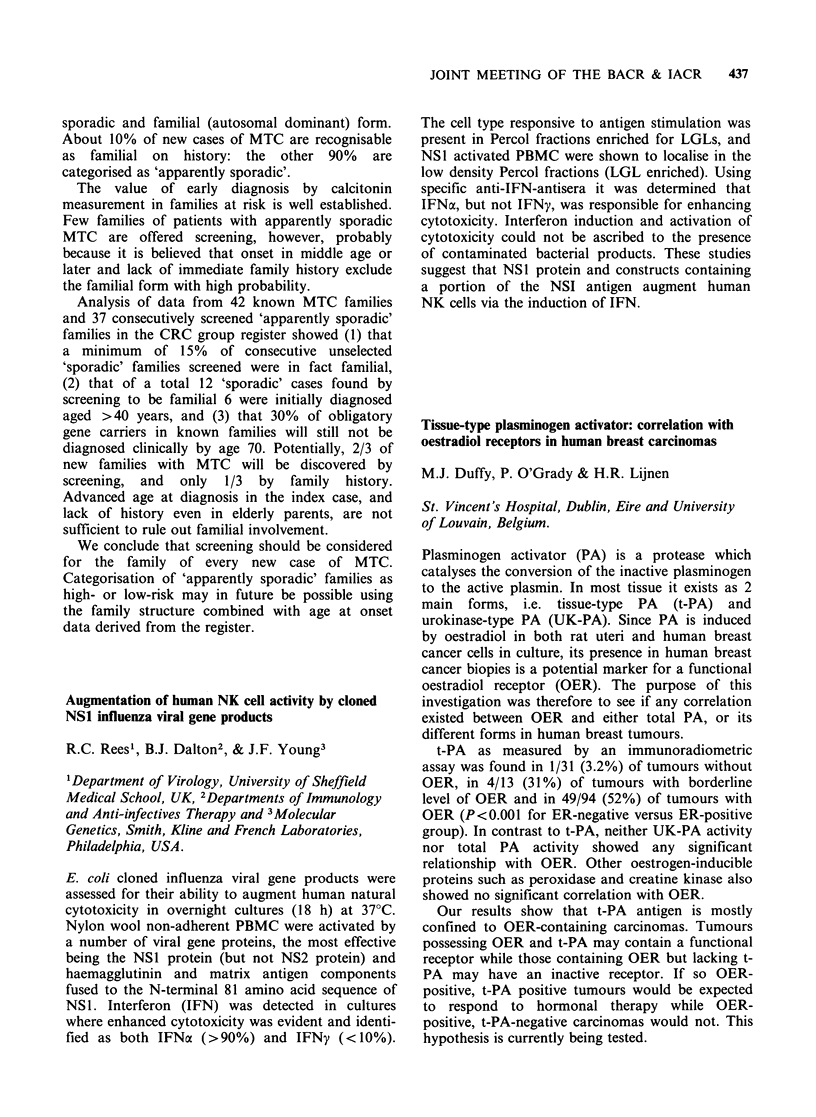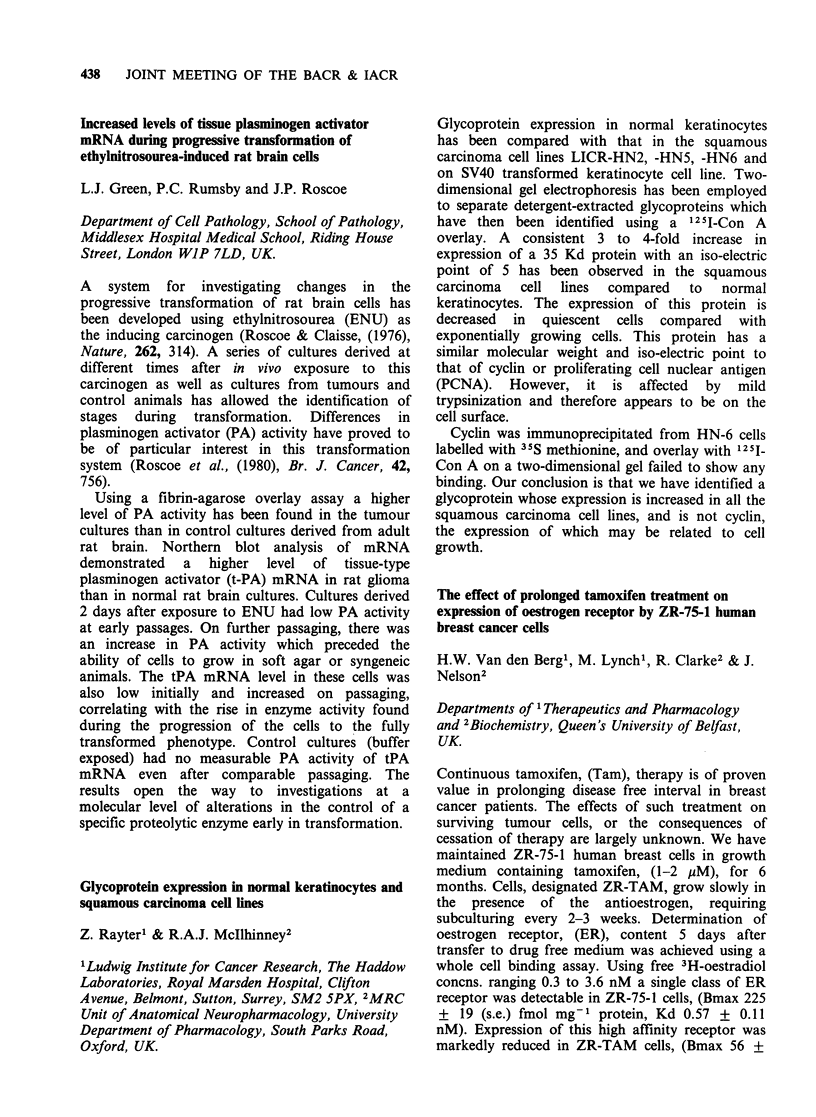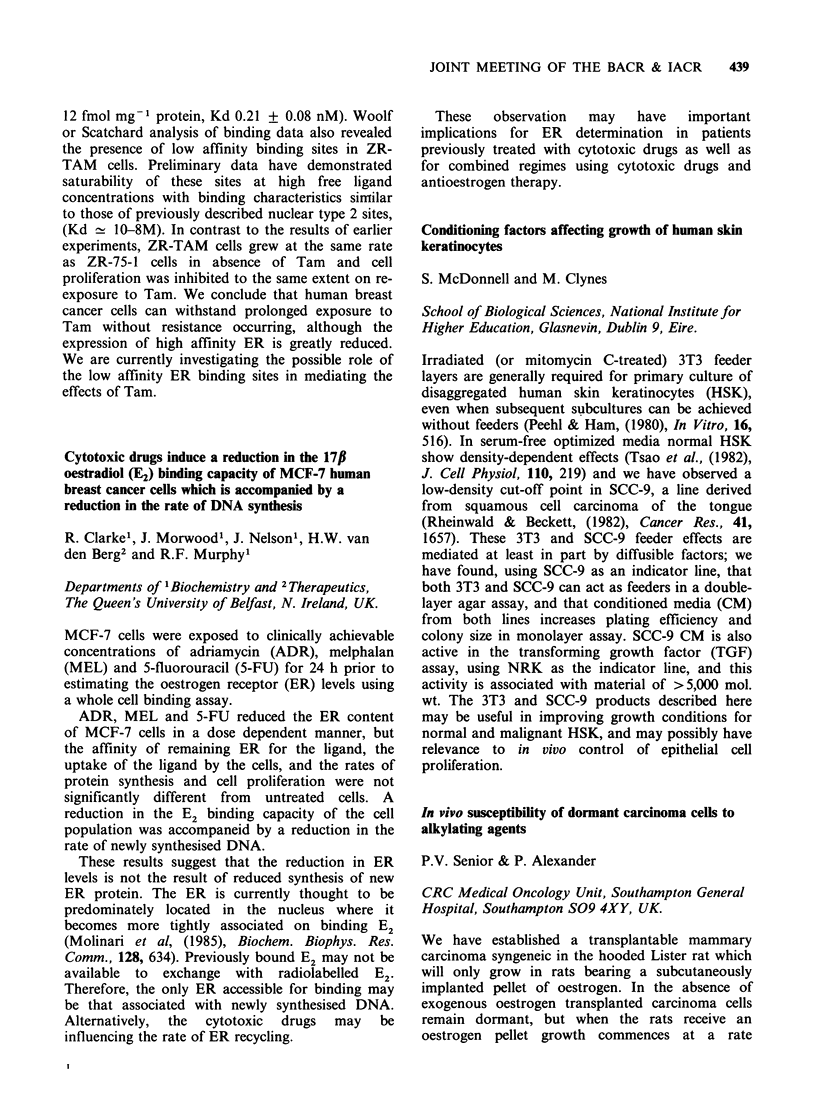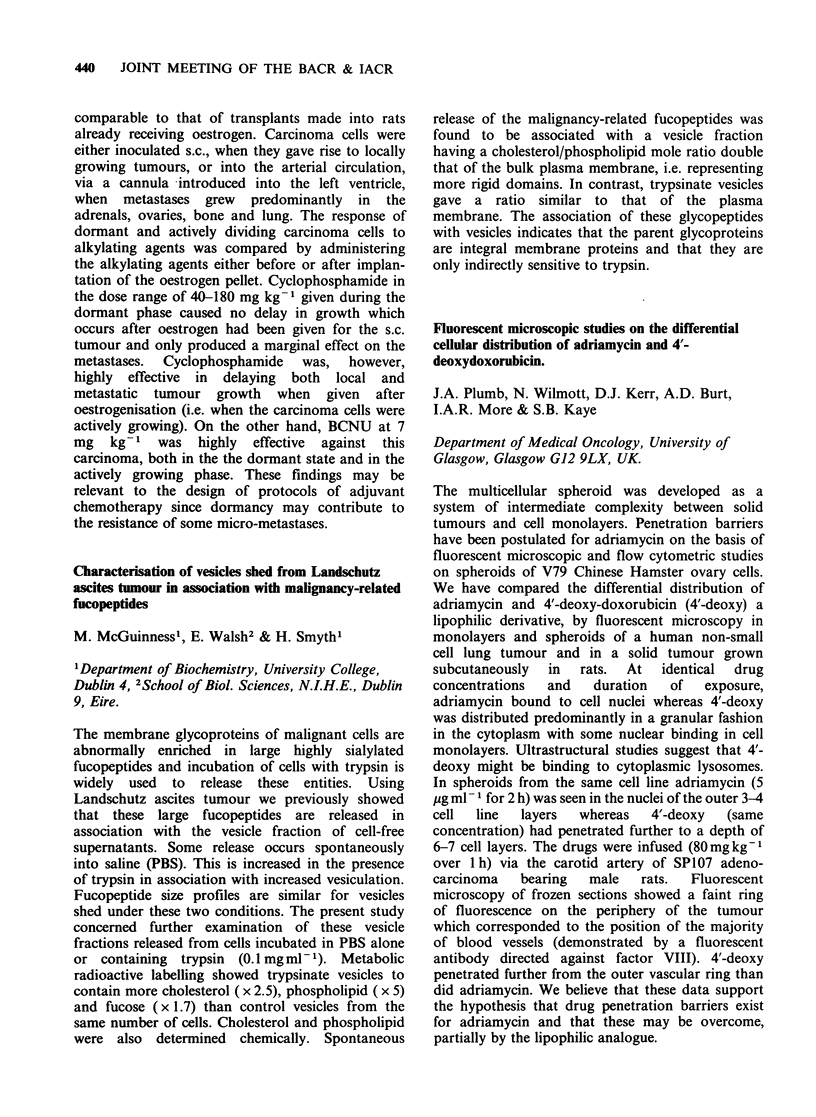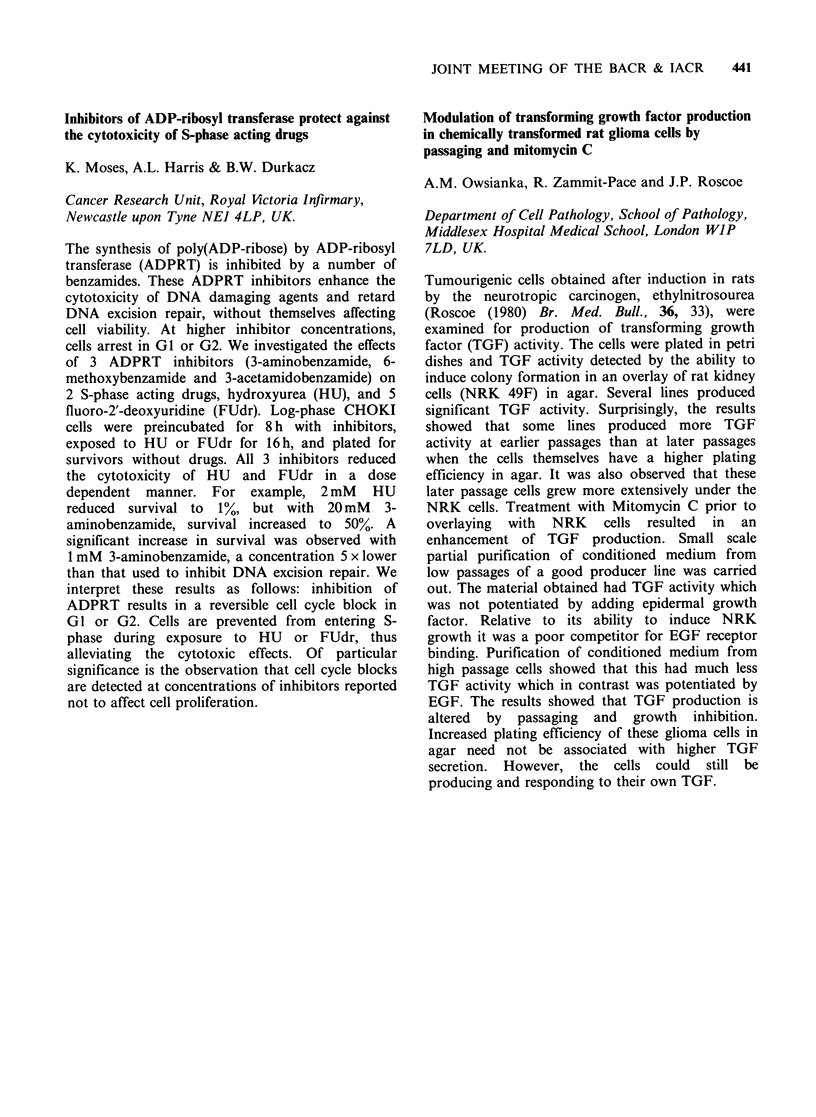# Joint meeting of the British & Irish Associations for Cancer Research. November 28-29, 1985, London. Abstracts.

**DOI:** 10.1038/bjc.1986.69

**Published:** 1986-03

**Authors:** 


*Br. J. Cancer* (1986), **53**, 419–441

### Joint meeting of the British & Irish Associations for Cancer Research*

(Incorporating a Symposium on 'Interferons as regulators of growth and function' and the Sixth Gordon Hamilton-Fairley Memorial Lecture†). November 28-29, 1985

*Held at the Royal College of Physicians, St Andrew's Place, London NW1*

### Abstracts of invited papers‡

### Regulation of gene expression by interferons

G.R. Stark, R.L. Friedman, M. McMahon, J. Kelly, S.P. Manly, A. Brasnett, A. Porter & I.M. Kerr

*Imperial Cancer Research Fund Laboratories, London, WC2A 3PX, UK.*

We have isolated cDNAs corresponding to several different mRNAs induced by $\alpha$-interferon (IFN) in human cells. Two code for metallothionein II (MT II) and a class I HLA; the others code for proteins of unknown sequence. The levels of all the mRNAs increase 5- to 40-fold about 1 day after treatment with IFN. Synthesis of new proteins is not required for induction. In IFN-resistant Daudi cells which retain IFN receptors, MT II, HLA and 2-5A synthetase mRNAs are induced normally, but several of the others are not induced. It seems likely that there are at least two pathways for induction, possibly reflecting a diversity of receptor function. In HeLa cells, most of the mRNAs are induced both by $\alpha$- and $\gamma$-IFNs, but at least one is preferentially induced by $\alpha$-IFN. The mRNA for c-*myc* decreases in response to $\alpha$-IFN but increases in response to $\gamma$-IFN. Thus, the control of IFN-inducible mRNAs is complex. The rate of transcription of the 1-8 mRNA, measured in nuclear run-off experiments, doubles 5 min after treatment with IFN, reaches a maximum by 60 min, and falls to the uninduced rate by 8–12 h. Activation of transcription also plays a major role in increasing the levels of MT II and HLA mRNAs in IFN-treated cells. In addition, from parallel measurements of rates of mRNA accumulation and transcription, it is clear that post-transcriptional events are also important in increasing the levels of IFN-induced mRNAs. When regions upstream of the human genes for MT II, 2 class I HLAs and one class II HLA are compared, a homologous sequence of ~15 base pairs is revealed. Similar sequences have also been found near the 5′ ends of IFN-induced mouse genes. The functions of these sequences are being characterised. The IFN-regulated human 6-16 gene, transfected into mouse cells, can be induced by mouse IFN. Therefore, it should be possible to conduct a detailed analysis of the promoter region.

*BACR enquiries to: BACR Secretariat, C/O Institute of Biology, 20 Queensberry Place, London SW7 2DZ, UK.

†This issue, pp. 301–306.

‡Reprints of these abstracts are not available – Ed.

### Interferon and HLA expression

M. Fellous & F. Rosa

*Department of Immunology, Pasteur Institute, 25, Rue du Dr. Roux, 75724 Paris Cedex 15, France*

Interferon (IFN) enhances cell surface levels of HLA class I and II histo compatibility molecules as well as $\beta_2$ microglobulin.

This increment follows the enhancement of the corresponding messenger RNA in the cytoplasm of cells analysed. Using isolated nuclei which allows direct quantification of the transcription rate of defined genes, one could show that IFN-$\beta$ and $\gamma$ enhance the transcription rate of HLA class I and $\beta_2$ microglobulin gene in a 3–4-fold ratio after 1 h of treatment. The enhanced transcription is maintained for at least 24 h. The transcription of HLA class II genes has also been analyzed. Interferon $\gamma$ appears to act as specific inducer of HLA class II genes.

Genetic control of IFN $\beta$ and $\gamma$ receptor has also been studied.

Finally the biological significance of HLA interferon regulation was discussed.

© The Macmillan Press Ltd., 1986

### Early and late events in the regulation of tumour cell proliferation by interferons

M.J. Clemens, R. Exley, V.J. Tilleray & N. Sharp

*Cancer Research Campaign Mammalian Protein Synthesis and Interferon Research Group, Department of Biochemistry, St. George's Hospital Medical School, London SW17 0RE, UK.*

A wide variation is observed in the sensitivity of both normal and tumour cells to the growth inhibitory and differentiation-inducing effects of interferons. In addition to possible differences in the levels of interferon receptors, such variation may be due to the nature of the signals generated by these receptors and in the responses to these signals of the cellular machinery involved in RNA and protein synthesis and in DNA replication. The key to understanding the molecular basis of cellular growth regulation by interferons lies in identifying the early signals and relating them to the subsequent changes which occur in gene expression, protein accumulation and DNA synthesis.

Our laboratory has concentrated on a detailed analysis of the changes in macromolecular synthesis which accompany interferon-induced impairment of cell proliferation. In the highly sensitive Burkitt lymphoma cell line, Daudi, growth inhibition is characterized by a decline in the rate of protein synthesis which does not involve the same mechanisms of translational control as those implicated in development of the interferon-mediated antiviral state. In parallel with the inhibition of protein synthesis, DNA replication is impaired by a mechanism which appears to involve deficient assembly of chromatin and instability of a substantial fraction of the newly replicated DNA. The relationships of these and other long-term changes to the more rapid effects of interferons, such as induction or repression of specific genes, modulation of cellular responses to growth factors, and changes in cellular or viral oncogene activity, are not yet understood.

### Interferons as immunomodulators

M. Moore

*Department of Immunology, Paterson Laboratories, Christie Hospital and Holt Radium Institute, Manchester, M20 9BX, UK.*

In addition to the induction of new RNA and protein and the inhibition of viral replication, the interferons (IFNs) can influence a multiplicity of cell types and functions. Since the cells of the immune system are among those so influenced, IFNs have been assigned an immunoregulatory role. IFN-$\gamma$ (immune IFN) plays an important part in the cascade of lymphokines generated during the adaptive immune response. Antigen-stimulated T cells produce IFN-$\gamma$ which in turn affects the responsiveness of T cells and is involved in the cooperation between T and B cells by acting as a B-cell maturation factor. In addition, IFN-$\gamma$ acts on the effector cells of the non-adaptive host response regulating proliferation, differentiation and function. Some of the effects of IFN-$\gamma$ are common to all types of IFN, but others are predominantly mediated by IFN-$\gamma$. Most of the multicellular effects of IFN$\gamma$, unlike those of IFN$\alpha/\beta$ are stimulating, rather than inhibitory, activating new genes. A recently recognised characteristic of IFN-$\gamma$ is its ability to act synergistically with other factors or cellular inducers. Some effects may not depend entirely on IFN-$\gamma$ *per se* but are possibly secondary to the IFN-$\gamma$-induced expression of surface receptors for other factors or regulatory substances. Studies of IFN-$\gamma$ contribute to our understanding of the regulation of the immune response and inflammation and may assist in clarifying some of the pathological processes involved in autoimmune and other disorders. Exploitation of the synergy between IFN-$\gamma$ and other cytokines could offer new prospects for the therapeutic application of these factors.

### Properties of interferon-$\alpha_2$ analogues produced from synthetic genes

V.E. Moore, R. Camble & M.D. Edge

*ICI Pharmaceuticals Division, Mereside, Alderley Park, Macclesfield, Cheshire, SK10 4TG, UK.*

We have shown that genes long enough to encode interferon-$\alpha_1$ and interferon-$\alpha_2$ can be synthesised chemically and expressed in *Escherichia coli.* In principle synthetic genes can be used to prepare analogues with any genetically coded amino-acid substituted at any number of desired positions throughout the length of the polypeptide chain. Using interferon-$\alpha_2$ as a model system we have been exploring the potential of this approach to generate protein analogues for structure-activity studies. Results to be presented illustrate that coherent structure-activity patterns are obtained for interferon analogues produced in this way. Some 20% of the sequence can be removed or extensively substituted without any great change in biological activity. Changes at just one or two residues conserved among all human interferon-$\alpha$ and interferon-$\beta$ species can greatly decrease or abolish biological activity. A dipeptide sequence has been identified which is critical for recognition by a widely available monoclonal antibody. We are convinced that the synthetic gene approach can produce target analogues with the throughput required to make useful progress in a protein structure-activity programme.

**Anticancer activity of interferons studied in animal tumour models**

F.R. Balkwill

*Imperial Cancer Research Fund, Lincoln's Inn Fields, London, WC2A 3PX, UK.*

Successful use of biological response modifiers such as interferons, (IFNs), in the therapy of cancer requires an understanding of their mechanisms of action. IFNs may act directly on tumour cells, suppressing growth, altering differentiated state or surface antigen expression, or by modulating host responses to the tumour. Using the nude mouse/human tumour xenograft model we have found that human IFN-$\alpha$s have a direct tumour inhibitory effect with no modulation of the murine host responses. Effects of IFN-$\alpha$s range from complete regression, through tumour stasis to progression at the same rate as control tumours and are consistent for each individual tumour line. Reasons for this difference are being investigated at the membrane receptor and molecular level. Human IFNs $\beta$ or $\gamma$ have little or no antitumour activity in this model system, but IFN-$\gamma$ stimulated levels of, or induced *de novo,* Class II HLA and tumour-associated antigens in some of the tumours, an effect which may have relevance in man.

Mouse IFN also inhibited the growth of human tumour xenografts, both resistant and sensitive to HuIFN-$\alpha$. Evidence so far would indicate that this was not due to a direct effect on the human tumour. Inhibition by murine IFN was also found in beige (NK deficient) nude mice suggesting that host NK cells were not involved in the inhibition. This model system therefore provides evidence of both direct and indirect effects of IFNs against cancer.

**Clinical and immunological studies of recombinant interferon gamma (immuneron$^{TM}$ Biogen) administered by 2 or 24 hour infusion in 30 patients with metastatic melanoma**

J.M. Kirkwood

*For The Yale Melanoma Unit and Biogen, Inc., USA.*

Thirty patients (pts) with advanced melanoma entered a phase I/II trial of IFN$\gamma$ at fixed dosages of 3, 30, 300, 1000, or 3000$\mu g m^{-2} d^{-1}$ x 14d unless dose-limiting toxicity (DLT) was encountered. IFN was administered either (a) by continous 24h infusion or (b) by daily 2h infusion: 11 pts with objective response or stable disease received maintenance IFN$\gamma$ at their original dosage for 1–6 cycles (14d/42d) until progression of tumour. Flu-like toxicity as with IFN$\alpha$ was seen in all dosages over 3$\mu g m^{-2}$ of IFN$_{\gamma}$. Neutropenia (not DLT) was seen in all dosages over 30$\mu g m^{-2}$: hepatotoxicity was DLT in 4 pts at 1–3000$\mu g m^{-2}$, and rigors/malaise were DLT in 2 pts at 3000$\mu g m^{-2}$. All toxicity was reversible after 24–96h off IFN$\gamma$. Complete response in lung disease was seen at 3$\mu g$, and partial response in lung disease at 300$\mu g m^{-2} d^{-1}$; both were maintained 6 mos(sched.B). Nine other pts received 1–6 mos. maintenance for stable/mixed responses. Serial study of oligo 2′ 5′ A synthetase, natural killer activity, and the phenotype of peripheral blood mononuclear cell in these pts. has revealed schedule and dose-dependent effects that are clustered in the dosage ranges between 300 and 1000$\mu g m^{-2} d^{-1}$ in both schedules A and B.

**Have interferons a clinical role in the management of patients with cancer?**

D. Crowther

*Department of Medical Oncology, Christie Hospital, Wilmslow Road, Manchester M20 9BX, UK.*

Although Interferon was discovered in the late 1950s by Isaacs & Lindenman, investigations of a possible clinical role were not possible until about 20 years later. Early studies of anticancer activity involved preparations from leucocytes with variable purity and potency, however, the recent cloning of human interferon genes and the application of recombinant DNA technology has resulted in the manufacture of pure forms of Interferon for clinical trial. Initial enthusiasm for treating patients with various forms of carcinoma has been tempered by the realisation that recombinant Interferon alpha used singly in current dose scheduling is of no value in the management of the more common forms of carcinoma (bronchus, breast, colorectal) but responses in patients with certain B cell malignancies have been more gratifying. 30-50% of patients with low grade lymphomas respond to Interferon alpha and in a series of previously untreated patients from Manchester it has been possible to postpone the necessity of using damaging and potentially carcinogenic cytotoxic drugs for about one year. The use of Interferon alpha in the treatment of patients with hairy cell leukaemia has been even more successful and this form of treatment is now first line therapy in the medical management of these patients.

The future holds further promise since new Interferons are now available for clinical testing and already there are suggestions that some patients with a particularly intractable malignancy (renal cell carcinoma) can have a useful response to Interferon gamma. The clinical role of combinations of bioregulators and their use in conjunction with chemotherapy remains largely unexplored but experimental studies indicate that this is an important avenue for further investigation. Phase I/II clinical trials of bioregulatory molecules pose difficulties which are more apparent than for cytotoxic drugs, since mechanisms of action are less well understood and the desired biological effect is unknown.

### Abstracts of members' proffered papers

### Induction and suppression of drug metabolizing enzymes by interferon in the mouse

D.J. Adams[1], S. Seilman[1], J.D. Hayes[2], F. Balkwill[2], B. Griffin[3] & C.R. Wolf[1]

*Imperial Cancer Research Fund [1]Lab. Mol. Pharmacology, Edinburgh, [2]Interferon Lab, Lincoln's Inn Fields, [3]Department Clinical Chemistry, University of Edinburgh, UK.*

The effect of murine interferon on drug metabolizing enzymes in the mouse has been studied because of the synergistic antitumour effects observed when interferon and chemotherapy are used in combination and also because of the potential effects of viral infection on the activation and deactivation of chemical carcinogens by these enzymes. Antibodies against rat or human glutathione transferases (GST) or cytochrome P-450s, which cross-react with mouse, were used. Western blots showed that proteins reacting with antibodies to GST subunits $Ya_1$ and $Yb_1$ were unchanged and those reacting with $Yb_2$, Yc, Yf, $GST_1$ and $GST_2$ were significantly suppressed. Interestingly protein equivalent to GST $\lambda$ which has been shown to be elevated in preneoplastic lesions, was significantly elevated by interferon-treatment. The metabolism of model GST substrates substantiated these findings where some activities were elevated, some unaffected and some suppressed. Similar data were obtained using the P-450 antibodies. A significant suppression of specific mono-oxygenase activities followed interferon administration.

### Effects of human α-2 interferon action on mutants of African green monkey kidney cells transformed by Simian virus 40.

H. Shine & J.K. Collins

*Virology Unit, Microbiology Department, University College, Cork, Eire.*

The CV-1 clone of African Green Monkey Kidney Cells is the permissive host of choice for Simian Virus 40 (SV40) replication. We have, using nitrosoguanidine mutagenesis isolated mutants of CV-1 cells which restrict SV40 replication. One such mutant clone designated $\mu 6$ has been selected for this study. These cells have the following properties:

(a) SV40 replication and cytopathic effect is restricted on superinfection.
(b) They express intranuclear 94 K T as well as two super T-antigens of 115 Kd and 125 Kd mol. wt.
(c) They contain very elevated levels of p53.
(d) They exhibit a transformed phenotype.
(e) They have integrated viral DNA.
(f) Wild type virus can be rescued at high efficiency on fusion with permissive cells.

Using infectious centre assays and *in situ* blotting we have found spontaneous release of low levels of wt infectious particles indicating slight breakthrough the mutant lesion. Using Sindbis virus challenge we found that treatment of $\mu 6$ cells with $10\,000\,\mu\,ml^{-1}$ of $\alpha$-2 interferon induced an antiviral state. This level of treatment did not affect the expression of 94 Kd T or the two super T-antigens. Interferon treatment however, did eliminate the spontaneous production of low levels of infectious virus found in untreated cells. Since these cells contain 94 Kd T-antigen which is refractory to interferon treatment interferon inhibition of SV40 in these mutant cells must be post transcriptional.

**The induction of MHC antigens on rat tumours by recombinant interferon-γ.**

H. Yeoman[1], R.A. Robins[1], R.W. Baldwin[1], P.H. Van der Meide[2] & H. Schellekens[2]

[1]*Cancer Research Campaign Laboratories, University of Nottingham NG7 2RD, UK, and* [2]*Primate Centre TNO, Rijswijk, The Netherlands.*

MHC encoded molecules play a pivotal role in the induction and regulation of immune responses. Interferons, most notably interferon-γ, have been shown to regulate MHC antigen expression. We have looked at the effect of rat recombinant interferon-γ on MHC antigen expression in cell cultures of a series of immunogenic and non-immunogenic, spontaneous and chemically induced rat tumours, by FACS analysis.

MHC class I can be augmented in 5/6 lines tested. Mc7 is a chemically induced immunogenic tumour that constitutively expresses high levels of class I, which can be augmented by interferon-γ to give up to a 7-fold increase. Sp15 and Sp22 are spontaneous non-immunogenic tumours that constitutively express lower levels of class I than Mc7, and can be augmented to express 3–4 times the level of antigen. Sp4, a spontaneous immunogenic tumour, expresses class I *in vivo*, but loses this expression when cultured *in vitro*. Class I expression is re-induced when the cell-line is incubated with IFN-γ. Induction of expression occurs as early as day 1 and persists at these levels until at least day 6. None of the tumours constitutively express, or have been induced to express, class II antigens, including the Y3 myeloma cell line.

The susceptibility of IFN-γ treated tumour targets to NK cytolysis has also been examined.

**Mechanisms of antitumour activity of IFN-A/D (BglI) on experimental metastases**

P. Ramani, I. Hart, F.R. Balkwill

*Imperial Cancer Research Fund, Lincoln's Inn Fields, London WC2A 3PX, UK*

A recombinant human hybrid alpha interferon (rIFN-αA/D) with antiviral, anti-proliferative and immunomodulatory effects on murine cells was used to treat mice with experimental metastases. There was significant inhibition of lung metastases 21 days after i.v. injection of $5 \times 10^4$ viable colon carcinoma, Colo 26, cells in mice treated with 500ng and 50ng daily 5 times a week. Similar results were seen in normal BALB/c, nude BALB/c and beige nude mice (NK cell deficient). Scheduling experiments *in vivo* showed that the most significant inhibition was seen when IFN was given only for the first 5 days although statistically significant reduction in lung tumour nodules and lung weights was seen when IFN treatment was initiated 7 days after the injection of tumour cells. rHuIFN-αA/D was cytostatic to Colo 26 *in vitro* causing 50% inhibition of cell growth or colony number with dose comparable to levels achieved in sera of mice. Although rHuIFN-αA/D stimulated NK cell activity in BALB/c mice, Colo 26 cells were resistant *in vitro* to NK cell lysis in both control and treated mice. All these results suggest that T cells and NK cells are not involved in the antimetastatic action of rIFN-αA/D.

The relative importance of IFNs effects on tumour cell proliferation, tumour cell surface antigen expression, and host, macrophage activity are currently being investigated. In this respect it is of interest that although Colo 26 cells are resistant to NK cells, they are lysed by activated macrophages. Further studies with spontaneous metastases of this tumour are also underway.

**Effect of interferon on the activity of cytotoxic agents in human lung cancer xenografts**

R.J. Fergusson, L.E. Anderson & J.F. Smyth

*Imperial Cancer Research Fund, Medical Oncology Unit, Western General Hospital, Edinburgh, UK.*

The effect of recombinant $\alpha_2$ interferon (IFN) on the activity of cis-platinum (CP) and ifosfamide (IFOS) was studied in human non small cell lung cancer xenografts grown in CBA mice rendered immunodeficient by neonatal thymectomy and total body irradiation. Groups of 6–9 tumours were randomised to single agent treatment, combination therapy (CP+IFN or IFOS+IFN) or a control group. IFN was injected s.c. in a dose of $2 \times 10^4$ IU daily for 35 days. Drugs were given i.p. weekly $\times 5$ at 20% of MTD (CP 1.4 mg kg$^{-1}$, IFOS 60 mg kg$^{-1}$). Tumours were measured three times per week and volume estimated assuming an ellipsoid shape. The median doubling time was calculated for each group and the activity of each agent or combination was expressed in terms of the specific growth delay (SGD) compared with controls. IFN showed no activity as a single agent (SGD<0.2) in these tumours. In a squamous carcinoma the activity of CP and IFOS alone (SGD=0.77 and 0.27) was increased by combination with IFN (SGD=1.44 and 0.66). A similar effect was seen in an adenocarcinoma (SGD for CP=0.12, CP+IFN=0.48, IFOS=0.04, IFOS+IFN=0.28) showing that IFN given in a dose which produces no cytotoxic effect alone is able to potentiate the effects of CP and IFOS in non small cell xenografts.

### The effect of recombinant γ interferon on the response to cytotoxic drugs of human lung cancer cells *in vitro*

P.R. Twentyman, N.E. Fox & N.M. Bleehen

*MRC Clinical Oncology and Radiotherapeutics Unit, Hills Road, Cambridge CB2 2QH, UK.*

A number of recent reports have indicated that, in both *in vivo* and *in vitro* model systems, α or β interferons can potentiate the cytotoxic or growth inhibitory effects of anti-tumour drugs. Following our studies of the effects of treatment with recombinant γ interferon (IFN-γ) on the growth of human lung cancer cells (Twentyman *et. al*, 1985, *Br. J. Cancer*, **52**, 21), we decided to investigate the influence of IFN-γ on the chemotherapy response of such cells. We used 3 cell lines with markedly different sensitivities to IFN-γ. NCI-H69 is a small cell line of low sensitivity, POC is a small cell line of intermediate sensitivity and COR-L23 is a large cell line of relatively high sensitivity. We have also examined the effect of IFN-γ on the drug response of a multi-drug resistant variant of NCI-H69 and the response to IFN-γ alone of a multi-drug resistant variant of COR-L23.

Using total cell number after 6–10 days as the response endpoint, the sensitivities to adriamycin (ADM), vincristine (VCR) and melphalan (MEL) were generally found to be unchanged in the presence of IFN-γ at lkU $ml^{-1}$ (NCI-H69 and POC) or 200 U $ml^{-1}$ (COR-L23). There was, however, a small degree of sensitisation by IFN-γ of POC to ADM, of COR-L23 to MEL and a small degree of protection of POC against VCR. These findings are currently being further quantified using clonogenic assay. Pretreatment of POC with IFN-γ for 48 h did not change the subsequent response to a 1 h treatment with ADM, VCR or MEL. A multidrug resistant variant of COR-L23 was more sensitive to IFN-γ alone than the parent line.

### Effects of recombinant interferon on the c-*myc* oncogene product in daudi cells

J. Geradts & K. Sikora

*Ludwig Institute for Cancer Research, MRC Centre, Hills Road, Cambridge, UK.*

The growth of human lymphoblastoid cells *in vitro* can be inhibited by interferon. It has previously been shown that this antiproliferative effect is accompanied by decreased transcription of the c-*myc* gene, the cellular homologue of the avian myelocytomatosis virus oncogene. As c-*myc* has been widely implicated in carcinogenesis, we have studied the modulation of the c-*myc* encoded protein ($p62^{c-myc}$) by recombinant interferon. To estimate $p62^{c-myc}$ levels, we used a recently developed monoclonal antibody raised against synthetic peptide fragments of this protein. Our experiments suggest that, in Daudi cells whose growth is arrested by interferon, $p62^{c-myc}$ levels are up to 75% lower than in control cells. There was correlation between the amount of c-*myc* encoded protein per cell and its proliferative status. Whether the reduction in the c-*myc* protein is the cause or consequence of the growth arrest induced by interferon is as yet unclear.

### Newly replicated DNA exhibits increased sensitivity to nuclease digestion in Burkitt lymphoma (Daudi) cells treated with human interferons

R. Exley & M. Clemens

*Cancer Research Campaign Group, Department of Biochemistry, St. George's Hospital Medical School, London SW17 0RE, UK.*

In Daudi cells, during inhibition of proliferation by human interferons, DNA synthesis becomes partially uncoupled from cell division. There is no accumulation of DNA in these cells but rather a rapid turnover of up to 50% of the newly synthesized DNA. This could be due either to increased cellular nuclease activity towards nascent DNA chains or to a greater susceptibility of newly replicated DNA to nucleases.

When control cells are labelled with $^{3}H$-thymidine and isolated nuclei are prepared, it can be shown that newly synthesised chromatin undergoes a series of structural changes which alter its sensitivity to micrococcal nuclease *in vitro*. Initially, newly labelled DNA is preferentially protected from digestion, perhaps because of association of replication forks with the nuclear matrix. Within 5–10 min this DNA transiently becomes more nuclease-sensitive, as the chromatin undergoes structural maturation. Within 20 min, the DNA acquires the nuclease-resistance characteristics of bulk parental chromatin. In contrast, in interferon-treated Daudi cells both at early and late times of labelling, the DNA in isolated nuclei shows increased susceptibility to nuclease digestion, suggesting that it is less protected by its association with histones or other proteins found in mature chromatin. No difference is observed in the nuclease sensitivity of bulk chromatin between control and interferon-treated cells, as measured by digestion of DNA labelled for 2 h, and normal nucleosome ladders are observed on agarose gel electrophoresis of the digestion products. These results suggest that DNA turnover in interferon-treated Daudi cells may be a consequence of defective assembly of newly replicated DNA into mature chromatin, perhaps because of an impairment in chromatin protein synthesis.

**Changes in cellular phenotype of Burkitt lymphoma (Daudi) cells associated with interferon-induced growth inhibition**

M. Clemens[1], R. Exley[1] & P. Knox[2]

[1]*Cancer Research Campaign Group,* [2]*Department of Biochemistry, St. George's Hospital Medical School, London SW17 0RE, UK.*

When Daudi cells are treated in culture with concentrations of human interferons within the physiological range, cell proliferation is inhibited and ceases completely after 2–3 days. The response is initially reversible and non-cytotoxic, but after 4–5 days cell viability decreases, possibly due to the inability of the cells to grow. Along with the changes in macromolecular synthesis described elsewhere there are modifications of cellular behaviour and surface properties suggestive of a 'less transformed' phenotype.

The cells become larger in size, although their DNA content does not increase. Analysis on a cell sorter indicates an incomplete accumulation in the G1 phase of the cell cycle. When placed in 5–10% serum-containing medium, interferon-treated Daudi cells show a marked enhancement of adhesion to tissue culture surfaces compared to control cells. This effect, which is similar to that seen with a variety of cell types after interferon treatment, can also be elicited by treatment with the phorbol ester TPA as well as by sodium butyrate and other inducers of cell differentiation. These results are compatible with the view that interferon treatment initiates a series of changes in expression of genes in Daudi cells which regulate not only cell proliferation but also affect plasma membrane properties and cytoskeletal organization. The latter changes may reflect partial differentiation of these transformed pre-B cells towards a state in which they show less tumourigenic and/or metastatic potential. Interferon-induced changes in the levels of receptors for exogenous growth or differentiation factors may be important in effecting this response.

**Regulation of protein synthesis in growth inhibited Burkitt lymphoma (Daudi) cells treated with human interferons**

V. Tilleray & M. Clemens

*Cancer Research Campaign Group, Department of Biochemistry, St. George's Hospital Medical School, London SW17 0RE, UK.*

Inhibition of proliferation of Daudi cells by human interferons is accompanied by a progressive decrease in the rate of cellular protein synthesis. We have investigated whether the mechanisms which control translation in interferon-treated, virus-infected cells are also responsible for the inhibition of protein synthesis in these uninfected cells.

The rate of polypeptide chain initiation is lower in interferon-treated Daudi cells relative to control cells, as indicated by a disaggregation of polyribosomes. This does not appear to be due to any inhibition of initiation factor eIF–2 activity since the ability of this factor to form [eIF-2.GTP.Met-tRNA$_f$] complexes or to bind Met-RNA$_f$ to native 40S ribosomal subunits in extracts from the cells remains unimpaired. There is also no change in the phosphorylation state of eIF-2 in Daudi cells following interferon treatment. No major decrease in the content of total mRNA in the cells can be observed up to 4 days of interferon treatment, as judged by the poly(A) content of purified cellular RNA and by the translatability of mRNA in cell extracts added to an mRNA-dependent reticulocyte lysate protein synthesizing system. Thus neither the dsRNA-activated eIF-2 protein kinase pathway (which would inactivate the initiation factor) nor the 2′5′ oligoadenylate-ribonuclease L pathway (which should inactivate or destroy cellular mRNA) appear to be involved in this system. Rather, there may be a block in polypeptide chain initiation at the level of mRNA binding to ribosomes to form functional 80S initiation complexes. These possibilities are currently under investigation.

### Enhancement of tumour cell malignancy by treatment with chemotherapeutic agents

T.J. McMillan, J. Rao & I.R. Hart

*ICRF Laboratories, Lincoln's Inn Fields, London, WC2A 3PX, UK.*

This study was undertaken to determine whether anti-cancer drugs, many of which are carcinogenic and mutagenic, could facilitate or promote tumour progression. Cells from the B16-F1 subline of the murine B16 melanoma were pre-treated with hydroxyurea, methotrexate or cytosine arabinoside prior to i.v. injection into groups of syngeneic mice in a series of individual experiments. Three weeks after injection mice were killed and the metastatic burden was evaluated by counting the number of lung tumour nodules. Treatment with all three agents brought about increases in metastatic capacity. Thus injection of $5\times10^4$ F1 cells pre-treated for 18 h with 0.1 mM or 0.3 mM hydroxyurea resulted in median numbers of 35 (range 19–72) and 31 (range 8–46) lung nodules respectively compared to the 2 (range 0–6) resulting from control cells. Similar results were achieved with nude mice. Incubation for 48 h with 500 nM or 750 nM methotrexate increased resultant lung tumour colonies from a median of 9 (range 2–27) to 30 (range 5–65) and 31 (range 15–52) respectively while 18 h incubation in 100 ng $ml^{-1}$ or 300 ng $ml^{-1}$ cytosine arabinoside produced 38 (range 15–56) and 5 (range 0–14) lung nodules respectively compared with 6 (range 1–9) for untreated controls. Treatment with all but the highest dose of cytosine arabinoside brought about a significant increase ($P<0.05$) in the metastatic abilities of these tumour cells and these results suggest that certain anti-neoplastic agents may play a direct role in facilitating tumour progression. Some of the factors which might be involved in this phenomenon, such as mutagenicity, alterations in NK cell sensitivity and cell cycle synchronisation have been examined.

### Biodistribution and tumour imaging characteristics of $^{131}I$, $^{123}I$ and $^{111}In$-labelled monoclonal antibody in mice with human tumour xenografts

M.V. Pimm[1], A.C. Perkins[2] & R.W. Baldwin[1]

[1]*Cancer Research Campaign Laboratories, University of Nottingham,* [2]*Medical Physics, University Hospital, Nottingham, UK.*

Monoclonal antibodies against human tumour associated antigens, radio-labelled with $^{131}I$, $^{123}I$ or $^{111}In$, are being evaluated for tumour imaging. Each of these radionuclides had different energies of $\gamma$-emission which will affect the quality of the images. In addition the radiometal $^{111}In$ will have a different biodistribution from that of radioiodide following antibody catabolism. This would also affect the imaging characteristics of the preparation. In the present study the biodistribution of $^{131}I$, $^{123}I$ and $^{111}In$ from labelled antibody (791T/36) have been compared in mice with human tumour xenografts.

Blood levels of radioiodine and $^{111}In$-labelled antibody were similar in relation to injected doses. However, whole body retention of $^{111}In$ was over twice that of radioiodine because radioiodide was excreted but $^{111}In$ was retained. Consequently, in relation to the whole body, blood levels of $^{111}In$ were lower than those of radioiodide. Xenograft localisation of $^{131}I$ and $^{123}I$ was virtually identical but the proportion of the injected dose of $^{111}In$ accumulated in tumour was up to five times higher. Higher levels and longer retention of $^{111}In$ gave tumour to blood ratios up to eight times those with $^{131}I$ or $^{123}I$. Gamma scintigraphy of tumour xenografts with $^{123}I$-labelled antibody was superior to $^{131}I$ due only to its lower energy of $\gamma$-emission. The energies of emission of $^{111}In$ are higher than $^{123}I$, but superior tumour localization of $^{111}In$ produced markedly better images than $^{123}I$-labelled antibody.

These studies indicate that the superiority of $^{111}In$ as a radiolabel for antibody imaging of tumours is due as much to its physiological fate as to its physical characteristics.

### Enzymic fragmentation of monoclonal antibodies to human tumour associated antigens: Biodistribution studies with $F(ab')_2$ and Fab in mice with human tumour xenografts

S.M. Andrew[1], L. Dale[1], M.V. Pimm[1], A.C. Perkins[2] & R.W. Baldwin[1]

[1]*Cancer Research Campaign Laboratories,* [2]*Medical Physics, University of Nottingham, UK.*

Monoclonal antibodies against human tumour associated antigens are being evaluated for both radioimaging of tumours and targeting of therapeutic agents. $F(ab')_2$ and Fab fragments may give greater tumour discrimination than intact antibody. In the present study the response of three monoclonal antibodies to enzyme treatment has been assessed and the tumour localization characteristics of their fragments determined.

Papain treatment of 791T/36 (IgG2b) yielded Fab fragments. $^{125}I$ labelled Fab localized in human tumour xenografts more rapidly than intact antibody but whole body survival of Fab was shorter than intact antibody. Digestion of 791T/36 failed to yield $F(ab')_2$, and fragments corresponding predominantly to Fabc were obtained in very low yield. With two anti-CEA antibodies (C/24 and 161, both IgG1) Fab and $F(ab')_2$ fragments were obtained. $^{125}I$ Fab and $F(ab')_2$ localized in xenografts more quickly than intact antibody. Higher tumour to blood (T:B) ratios were seen with fragments (T:B = 10:1) than with intact antibody (T:B = 3:1). However, whole body survivals of Fab and $F(ab')_2$ ($t_{1/2} = 8$ h and 14 h respectively) were shorter than intact antibody ($t_{1/2} = 75$ h) and this produced lower absolute levels in tumour. Gamma scintigraphy with $^{131}I$ labelled fragments gave earlier and superior imaging of tumours than did $^{131}I$-intact antibody but this was most marked with the Fab fragment.

Generally fragments do give more rapid and superior tumour localisation than intact antibody, and this gives more favourable gamma camera imaging. Absolute levels in tumour are lower than with intact antibody and this should be appreciated in considering conjugates of fragments and drugs for targeted therapy.

### Preliminary evaluation of monoclonal antibodies to oncogene products as drug-targeting vectors

M.J. Embleton[1], M.C. Garnett[1], N.A. Habib[2] & C. Wood[2]

[1]*Cancer Research Campaign Laboratory, University of Nottingham, Nottingham NG7 2RD and* [2]*Department of Surgery, Royal Postgraduate Medical School, London W12 0HS, UK.*

Monoclonal antibodies to $p28^{sis}$ and $p21^{ras}$ have been studied as potential anti-tumour drug-targeting agents using a panel of cultured human tumour cell lines. Both antigens were detected at the cell membrane by immunofluorescence on fixed cytocentrifuge preparations, virtually all cells exhibiting strong staining. Viable cells in suspension or cytocentrifuge preparations fixed after staining were largely negative, <5% of cells showing fluorescence. This was confirmed by flow cytofluorimetry, which gave a high signal for fixed cells but a low signal for viable cells. These results are consistent with the distribution of the oncogene products on the inside of the cell membrane rather than the outside.

Anti-$p21^{ras}$ was linked to human serum albumin conjugated to rhodamine in order to determine the internalisation potential of anti-$p21^{ras}$ vectored compounds into cultured tumour cells. The anti-$p21^{ras}$ conjugate showed minimal surface binding and only trace amounts of rhodamine appeared in the cytoplasm over 4 h incubating at 37°C. By comparison, a similar conjugate prepared with another antibody, 791T/36, resulted in strong cytoplasmic labelling in the perinuclear area. A drug conjugate prepared by linking anti-$p21^{ras}$ to methotrexate-substituted human serum albumin (MTX-HSA) was only marginally more cytotoxic to tumour cells than MTX-HSA alone, and 50–100 times less toxic than free methotrexate. In comparison, a similar conjugate prepared with the same batch of MTX-HSA but antibody 791T/36 was highly toxic to relevant target cells. Monoclonal antibodies to oncogene products do not appear to be suitable as anti-tumour targeting agents.

### Is production of human chorionic gonadotrophin (hCG) or alpha fetoprotein (AFP) by somatic tumours a marker of chemosensitivity?

S.M. Crawford, J.A. Ledermann, G.J.S. Rustin, R.H.J. Begent, E.S. Newlands & K.D. Bagshawe

*Department of Medical Oncology, Charing Cross Hospital, London W6 8RF, UK.*

Some tumours of somatic origin have elements within them which produce markers which are associated with germ cell or gestational trophoblastic tumours. This biochemical behaviour may be associated with trophoblastic differentiation on histology. In our laboratory, hCG is elevated in 19.6% of samples from patients (pts) with upper gastrointestinal (GI) tumours and 19% from those with bladder tumours. (AFP 14.4% and 13% respectively.) We have treated 7 pts with GI and bronchial tumours with the high risk choriocarcinoma regimen (etoposide 100 mg $m^{-2}$ (E) plus actinomycin D 0.5 mg. both day 1 and 2, methotrexate 300 mg $m^{-2}$ day 1 followed by folinic acid rescue (MTX), alternating weekly with cyclophosphamide 600 mg $m^{-2}$ plus vincristine 1 mg $m^{-2}$ (VCR)) with these results; 2 tumours producing AFP achieved biochemical PR; 5 tumours producing hCG, 4 biochem CR, 1 early death. Two patients (1 Ca bladder, hCG; 1 Ca bronchus, hCG+AFP) were treated with the relapsed germ cell tumour regimen (E plus cisplatin 100 mg $m^{-2}$ alternating after 8–10 days with MTX, VCR and bleomycin 30 mg 48 h infusion). Both achieved biochem CR. All biochem CR were accompanied by anatomical PR or CR.

These responses may reflect particular sensitivity in the marker producing tumours or show that treatment of common tumours requires chemotherapy of this intensity. Pts with common solid tumours should have the serum hCG and AFP measured so that this approach can be applied to larger numbers.

### DD9-E7 and other epithelial markers in adenocarcinomas of the exocrine pancreas

E. Heyderman[1], S. Larkin[1], J. Hermon-Taylor[2] & A. Grant[2]

[1]*Department of Histopathology, UMDS, St. Thomas Hospital, London SE1 7RQ,* [2]*Department of Surgery, St. George's Hospital, London SW17 0RE, UK.*

The identification of the primary site of an adenocarcinoma which presents with metastatic disease and no localising signs and symptoms is a major problem in routine surgical histopathology. In some cases, as with metastatic prostatic or thyroid carcinoma, markers such as prostatic acid phosphatase and thyroglobulin, which have a high degree of specificity, are available for immunocytochemical staining of fixed tissue sections. As yet there do not appear to be specific markers for tumours of the gastrointestinal tract, which are a common source of metastatic deposits. While it is often possible to demonstrate primary lesions in the intestinal tract by barium studies, the diagnosis of pancreatic adenocarcinoma by a variety of methods, including CAT-scan, may be very difficult. Our approach has been to evaluate an ascites preparation of a mouse monoclonal antibody, DD9-E7, directed against a pancreatic tumour line GER, and compare it with monoclonal antibodies to carcinoembryonic antigen (CEA), epithelial membrane antigen (EMA) and cytokeratin (CAM 5.2), using an indirect immunoperoxidase technique on 22 primary pancreatic adenocarcinomas. Some cases were resection specimens, while others were open pancreatic needle biopsies. All 22 were positive for DD9-E7, EMA and CAM 5.2. 20/22 were positive for CEA, but often weakly and focally, in sharp contrast with gastric or colorectal carcinomas which are usually strongly positive. If further work with DD9-E7 supports this high positivity in pancreatic tumours, its combination with CEA would be useful in discriminating from tumours from other sites, only some of which are CEA and/or DD9-E7 positive.

### The quantitation of the c-*myc* oncogene product in testicular and cervical neoplasia

J.V. Watson[1], H. Cox[1], G. Evan[1], P. Hendy-Ibbs[1], C. Munn[1], J. Stewart[1], K. Sikora[2]

[1]*MRC Clinical Oncology Unit, and* [2]*Ludwig Institute for Cancer Research, MRC Centre, Hills Road, Cambridge, UK.*

A simultaneous flow cytometric assay for the c-*myc* oncoprotein and DNA in nuclei extracted from archival paraffin wax embedded clinical biopsies is presented. Nuclei were extracted by pepsin digestion after dewaxing 20 μm sections. The c-*myc* oncoprotein was probed with a mouse monoclonal antibody, Myc 1-6E10, raised against a synthetic peptide. The latter corresponded to a hydrophilic region of the protein predicted from amino acids 171–188 derived from the base sequence of the cloned gene.

Normal cervical biopsies exhibited raised c-*myc* oncoprotein levels as determined by this antibody when compared with biopsies of cervical cancer. Carcinoma *in situ* specimens exhibited two subsets, one with high and one with low oncoprotein content. Preliminary results with cervical brushings from the colposcopy clinic show similar trends. These findings are potentially significant for mass cervical screening as up to 500 specimens could be examined per day with the Cambridge MRC flow cytometer.

The nuclear DNA and $p62^{c-myc}$ content of 43 primary testicular tumours was analysed. There was good correlation between the histological assessment, staining intensity and the levels of nuclear $p62^{c-myc}$ obtained. Those with elevated levels (mean 513 units) of $p62^{c-myc}$ remained alive and well at 5 years. Those with low levels (<mean 155 units) died or developed recurrence ($P<0.001$). The assay of $p62^{c-myc}$ in testicular tumours may have prognostic value.

### Clinical significance of the c-*myc* oncogene product in patients with solid tumours

S.Y.T. Chan, H. Gabra, F. Hill, G. Evan & K. Sikora

*Ludwig Institute for Cancer Research, MRC Centre, Hills Road, Cambridge, UK.*

We have studied the clinical applications of a set of monoclonal antibodies raised against synthetic peptides constructed from sequence data for the human c-*myc* oncogene product. One antibody, mycl-9E10, raised against the c-terminal 32 amino acids, has been shown to detect the 62,000 dalton c-*myc* gene product ($p62^{c-myc}$) in colo 320 cells. Western immunoblotting of sera and urine with this antibody consistently revealed a single 40,000 dalton band (p40). Quantitative analysis using dilution dot immunoblotting demonstrated an over three-fold increase in the titre of p40 in the sera of patients with a wide range of solid tumours ($n=43$), when compared to both healthy controls ($n=20$) and patients with non-malignant diseases ($n=25$). Peptide mapping by limited proteolytic analysis of p40 failed to confirm co-identity with $p62^{c-myc}$ in colo 320 cell lysate. These data suggest that p40 may be a novel tumour marker.

Radiolabelled antibody was injected i.v. into 14 patients with primary lung cancer and 6 patients with lung metastases from tumours arising in different organs. There was selective uptake at the primary tumour site of 12 patients with carcinoma of the bronchus suggesting a large quantity of the $p62^{c-myc}$ in these areas. Metastases derived from bronchial carcinoma did not take up the antibody. The patients with pulmonary metastases arising from sites other than lung also failed to take up antibody. Successfully localized tumours were all 3 cm or more in diameter. Such antibodies may be of use in monitoring tumour load and response to therapy.

### Predicting clinical outcome of prostate cancer by tumour cell DNA content.

M.V.P. Fordham[1], A. Burdge[2], J. Mathews[1], G. Williams[2] & T. Cooke[1]

*Departments of [1]Surgery, [2]Urology, [3]Histopathology, Charing Cross, and Westminster Medical School, London W6 8RF, UK.*

All patients from 1972 to 1983 with prostate cancer who underwent prostatectomy prior to treatment have had tumour cell DNA content measured by flowcytometry and microdensitometry and this correlated with clinical outcome. Tissue was formalin fixed, sectioned and graded histologically by the Gleason system. A Vickers micro-densitometer was used to measure tumour cell DNA content in Feulgen stained 6 $\mu$m sections, benign cells acting as controls. Cell suspensions were prepared from 40 $\mu$m sections by pepsin dis-aggregation and then stained with the fluorochrome propidium iodide. Fluorescence was measured using a FACS II flowcytometer for 30,000 cells and the histogram classified as aneuploid or diploid by comparison with benign cells.

One hundred and sixty four (90%) of patients had adequate tissue available and had a known outcome. Forty five (26.4%) tumours were aneuploid with an average age at diagnosis of 71.5 $\pm$9.5 (range 48–97) and 119 diploid age 71.7$\pm$7.3 (range 53–93). At diagnosis, 73% of aneuploid lesions had local or distant spread compared to 50% of diploid cancers. The average Gleason grade for aneuploid tumours was 7.0$\pm$1.7 (range 4–10) and diploid 6.1$\pm$1.9 (range 2–10). Despite any treatment, survival was worse in the aneuploid group ($\chi^2=21.3$, $P<0.001$) with 75% of the patients dead at 5 years, 90% from their cancers compared to 20% of the diploid group.

We conclude that patients with aneuploid prostate cancers are more likely to have advanced disease and die earlier from their cancers.

### Flow cytometric analysis of the DNA content of gastric cancer

K.C. Ballantyne, P.D. James, R.A. Robins, R.W. Baldwin & J.D. Hardcastle

*Departments of Surgery and Histopathology, University Hospital, Nottingham, and Cancer Research Campaign Laboratories, Nottingham, UK.*

Abnormal tumour cell DNA content (aneuploidy) is associated with worse prognosis in a variety of cancers and in a recent Japanese study only 17/54 (32%) gastric cancers were aneuploid.

Seventy-seven consecutive patients, median age 67 years (43–88 years) who underwent gastrectomy between 1979–1982 were studied. DNA content was measured by flow cytometry after disaggregating representative paraffin embedded sections (2 x 30 $\mu$m) and staining with diamidinophenylindole hydrochloride. 48 (62%) had a significant population of cells (>5%) with an abnormal DNA content (aneuploid). Two separate tumour blocks were examined in 24 cases and concordance found in 19 (79%). No correlation was found between DNA content of primary tumours and histological type, histological grade or pathological stage. Data from 44 patients surviving curative resection were analysed. 19 (43%) survived over 2 years and 10 remain disease free. The median survival was 23 months (6–67 months) for diploid tumours (n = 16) and 17 months (4–66 months) for aneuploid tumours (n = 28).

We conclude that factors other than tumour cell DNA are responsible for the aggressive nature of gastric cancer. Only 38% of cancers studied were diploid compared with 68% of tumours in Japan; this may reflect a difference in the geographical pattern of this disease.

### Sialic acid levels in peripheral and tumour-draining blood in carcinoma of the colon

P. O'Byrne[1], R. Browne[2], A. Johnson[2], P. Collins[2], J. Duignan[1] & D. Bouchier-Hayes[1]

[1]*Department of Surgery and* [2]*Department of Biochemistry, Royal College of Surgeons in Ireland, Dublin 2, Eire.*

Alterations in the levels of sialic acid in the peripheral blood of cancer patients have been reported but its role as a tumour marker is controversial. In this study, levels of sialic acid were measured in two blood samples drawn simultaneously from patients undergoing surgery for carcinoma of the colon. Peripheral bood (PB) was taken from the cubital vein and a tumour-draining blood (TDB) sample was obtained peri-operatively from a colic vein. 35 patients were investigated of whom 16 were classified as Duke's stage A and B and 19 as Duke's stage C and D. Tumour invasiveness, with involvement of extra colonic tissue, was assessed at the time of operation. Sialic acid was measured in serum samples using an enzymatic coupling procedure (Boehringer Mannheim Biochemica). The incidence of raised sialic acid levels was higher in TDB (68.8%) than in PB (50.0%) in patients at Duke's stages A and B, but was lower in Duke's stages C and D patients, 79.0% compared to 84.2% respectively. When sialic acid in the two blood samples from individual patients was compared, the ratio of sialic acid in PB to that of TDB increased significantly ($P<0.05$) with cancer stage. The ratio (PB/TDB) in Duke's A/B patients was 0.92 and in patients staged as Duke's C/D was 1.16. In patients with invasive tumours ($n=11$) this ratio increased to 1.40. Thus it would appear that comparing sialic acid levels in blood from these two sites might constitute a useful indicator of metastatic tumour spread and a possible means of assessing the extent of tumour invasiveness in colon cancer.

### Preferential expression of tumour associated antigens by aneuploid and clonogenic tumour cells

L.G. Durrant[1], R.A. Robins[1], N.C. Armitage[2], J. D. Hardcastle[2] & R.W. Baldwin[1]

[1]*Cancer Research Campaign Laboratories, University of Nottingham, NG7 2RD, and* [2]*Department of Surgery, Queens Medical Centre, Nottingham NG7 2UH, UK.*

As heterogeneity of antigen expression may prove a problem in using monoclonal antibody-drug conjugates in treatment of colorectal tumours, populations of tumour cells recognised by monoclonal antibodies have been analysed. Carcinoembryonic antigen recognised by monoclonal antibodies L11/265, C24 and 161 and the difucosylated blood group H determinant recognised by monoclonal antibody C14 were both preferentially expressed in aggressive aneuploid rather than diploid tumours. Furthermore if the cells from aneuploid tumours were stained with monoclonal antibodies C161, C24, C14 or 791T/36 and sorted on a FACS IV, there was an accumulation of aneuploid cells in the antigen positive population and a decrease in the antigen negative population. The enrichment of aneuploid cells by monoclonal antibody staining and cell sorting was directly related to both the number of cells within a tumour which stain and the amount of staining per tumour cell. Similarly if antigen positive and negative cells from colorectal tumours were sorted and analysed for their ability to incorporate $^{75}$Se-selenomethionine in a 24 h growth assay, the antigen positive cells incorporated 3–6 times more label than the antigen negative cells. Tumours cells isolated by growth in soft agar express all three antigens, including p72–791T, an antigen expressed weakly in the primary tumours. Despite antigenic heterogeneity in tumours antibody mediated drug therapy may be useful in treatment of colorectal cancer as selective monoclonal antibodies recognise aggressive aneuploid and rapidly dividing tumour cells.

### Cis platinum/chlorambucil in epithelial ovarian carcinoma

R.J. Atkinson

*Department of Oncology, Whitla Medical Building, Queen's University, Belfast, BT9 7BL, UK.*

The annual death rate from ovarian cancer in England and Wales is in excess of 4,000. This is greater than the total from cervical and uterine cancer.

The treatment policy of the joint gynae/oncology unit at the Belfast City Hospital is debulking surgery followed by chemotherapy.

From 1980 to 1983, 41 patients were treated with a combination of cis platinum 20 mg m$^{-2}$ and chlorambucil 0.15 mg kg$^{-1}$ for 5 days on each of 5 courses. Five years have elapsed since entry of the first patient. One patient died of myocardial infarction, leaving 40 evaluable patients. Twenty-three Stage III/IV patients had disease remaining after surgery. Of these, eight (34%) had a complete response, two (8.7%) had a partial response making a total response rate of ten (43%). Thirteen (57%) showed no response.

Currently there are 11 patients alive. The median survival time of Stage I and II patients is 54+ months while that for Stage III and IV is 21 months ($P<0.0114$).

### A prospective study of alternative response criteria for bone metastases from breast cancer

R.E. Coleman[1], G. Mashiter[2], K.B. Whitaker[3], D.W. Moss[3], I.S. Fogelman[2], R.D. Rubens[1]

[1]*ICRF Breast Unit and* [2]*Department of Nuclear Medicine, Guy's Hospital, and* [3]*Department of Clinical Pathology, Hammersmith Hospital, London, UK.*

Assessment of response in bone metastases is difficult as radiological evidence of healing may not be evident for many months. The roles of biochemical indices of bone metabolism, bone scanning and symptomatic response were prospectively studied in 53 patients receiving a total of 68 treatments. 47 patient treatments are currently evaluable.

Fifteen patients achieved a partial response (PR) by UICC criteria, 10 showed no change (NC) for at least 3 months and 22 had progressive disease (PD). Bone healing was associated with a transient increase in osteoblast activity (flare) with a rise at 1 month in alkaline phosphatase bone isoenzyme in 15/15 and osteocalcin in 13/15, followed by a fall in both towards normal. Eight of the 15 PRs showed evidence of a flare response on the bone scan, characterised by increasing activity of base-line lesions and occasional apparent new lesions at 3 months, followed by improvement at 6 months. Response in bone was associated with a fall in urinary calcium excretion (13/15) and symptomatic improvement (12/15). Symptomatic deterioration, increasing urinary calcium excretion, hypercalcaemia and declining osteoblast activity were only seen with PD. NC patients usually had symptomatic and biochemical improvement particularly when NC persisted for 6 months. Biochemical and symptomatic assessment of patients with bone metastases can predict objective response to systemic therapy.

### Metozantrone (M) *vs.* Adriamycin (A) in combination chemotherapy for advanced breast cancer

R.C.F. Leonard[1], M.A. Cornbleet[1], S.B. Kaye[2], A.W. Hutcheon[3], M. Soukop[2], S.M. Robinson[4] & J.F. Smyth[1]

*Departments of Oncology,* [1]*Edinburgh,* [2]*Glasgow &* [3]*Aberdeen, UK, and* [4]*Scottish Cancer Trials Office, Edinburgh, UK.*

Having previously demonstrated the activity of M in patients with advanced breast cancer (*Eur J. Cancer Clin. Oncol.*, (1984), **20**, 1141), we are now comparing M with A in a multicentre trial, both drugs being combined with vincristine (V) and prednisolone (P). To date, 115 patients have been randomised to receive either VAP (V 1.4 mg m$^{-2}$, A 50 mg m$^{-2}$, P 40 mg od × 5 d) or VMP (V 1.4 mg m$^{-2}$, M 14 mg m$^{-2}$, P 40 mg od × 5 d) each given q 3/52 × 3, 6, 9 or 12 courses depending on response. 76 patients are analysed for response to first line treatment. The trial closes when a minimum of 100 patients have completed 2 treatment courses. Patients with progressive disease are eligible for cross-over.

| | N | CR | PR | NR | |
|---|---|---|---|---|---|
| VAP | 38 | 5 | 18 | 15 | Overall response higher for VAP |
| VMP | 38 | 1 | 12 | 25 | $P=0.02$ |

At all evaluable sites, the response rates reflected the slightly higher activity of the VAP regimen. The median response duration to VAP was 8 months and VMP 5.5 months. However the survival patterns are identical. Assessed over 3–6 courses, VMP was clearly less toxic in terms of nausea and vomiting and especially alopecia. Significant falls in cardiac ejection (stress) fractions were seen for both regimens within the recommended accumulated doses but without clinical toxicity. Thus the more active and clinically more toxic VAP combination conveys no survival benefit in patient populations which were well matched in terms of the usual prognostic indicators.

### Adjuvant chemotherapy with cyclophosphamide, methotrexate and fluorouracil (CMF) in early breast cancer: Mechanisms of action

N. Padmanabhan[1], A. Howell[2] & R.D. Rubens[1]

[1]*ICRF Breast Cancer Unit, Guy's Hospital, London SE1 9RT, UK, and* [2]*CRC Department of Medical Oncology, Christie Hospital, Manchester, M20 9BX, UK.*

Adjuvant CMF in premenopausal patients (*pre* pts) with early breast cancer increases relapse free survival (RFS) and induces permanent ovarian ablation in 70% by 6 months. The contributions of ovarian ablation and direct tumoricidal effect of CMF need to be defined. For this purpose, the relation between estrogen (ER) and progesterone (PR) receptor status, RFS and CMF was studied in 407 pts, *pre:* 107 and 100, controls and CMF respectively, and postmenopausals (*post*): 101 and 99, entered into a randomized adjuvant CMF trial. Median follow up 36 and 40.5 months, *pre* and *post* respectively. *Results:* In *pre* ER but not PR, was strongly associated with a longer RFS in controls while for pts on CMF PR was strongly and ER less strongly associated with a longer RFS. In *post* there was no association between ER, PR, CMF and RFS. On analyses of subgroups of *pre* longer RFS and survival with CMF compared to controls was confined to PR+ and ER+ PR+ subgroups. There was no difference in RFS between controls and CMF for ER− PR− subgroup of *pre* and in all *post* subgroups. *Conclusion:* The alteration of the relation between receptor status and RFS in *pre* by CMF, longer RFS with CMF being confined to *pre* with PR+ tumours, no prolongation of RFS in ER− PR− *pre* (pts in this subgroup unlikely to respond to ovarian ablation) and in *post* (CMF has no intrinsic hormone activity and does not change the levels of steroids and gonadotrophins) given CMF, suggest that much of the effect of adjuvant CMF in *pre* is due to ovarian ablation.

### High dose carboplatin and autologous bone marrow infusion

C.J. Gallagher, M. Gore, I.E. Smith, S.J. Harland & E. Wiltshaw

*Department of Medicine, Royal Marsden Hospital, London SW3, and Sutton, Surrey, UK.*

Carboplatin (JM8) has proved to be as effective as cisplatinum in ovarian cancer and to be active in small cell lung cancer. The dose limiting toxicity has been myelotoxicity without the neuro- or nephro-toxicity associated with cisplatinum. We have therefore investigated the use of increasing doses of JM8 with autologous bone marrow infusion (ABMI) in a selected group of poor prognosis patients with ovarian or small cell lung cancer. In half the patients receiving 650 mg $m^{-2}$ or more, bone marrow harvested under general anaesthetic and $2 \times 10^8$ nucleated cells $kg^{-1}$ returned 16h after treatment or cryopreserved and returned after subsequent courses.

Myelotoxicity was unaffected by the use of autologous bone marrow infusion following JM8 at 650 mg $m^{-2}$ and 800 mg $m^{-2}$. Up to 5 courses per patient have been given at 650 mg $m^{-2}$ without ABMI but so far no patients have received more than two courses at 800 mg $m^{-2}$. There has been no clinical neurotoxicity, but two patients have shown a fall in glomerular filtration rate.

| *Dose mg m$^{-2}$* | *Patients* | *Courses* | *With ABMI* | *Myelotoxicity* | |
|---|---|---|---|---|---|
| | | | | *WBC* | *Platelets* |
| 520 | 5 | 17 | 0 | 2.4–4.4 | 42–220 |
| 650 | 6 | 15 | 5 | 2.4–7.6 | 63–394 |
| 800 | 6 | 10 | 5 | 0.4–3.2 | 9–47 |
| 1000 | 1 | 1 | 1 | 0.8 | 19 |

### Alpha lymphoblastoid interferon for non-invasive bladder cancer

R.T.D. Oliver[1,2,3], J.H. Waxman[1,2,3], H. Kwok[3], C.G. Fowler[2], P. Mathewman[3] & J.P. Blandy[2,3]

[1]*The London Hospital,* [2]*St. Bartholomew's Hospital, and* [3]*The Institute of Urology, London, UK.*

Sixteen patients with recurrent bladder cancer (8 with multiple papillary recurrences and 8 with flat-in-situ disease) were treated with topical alpha lymphoblastoid interferon. Each patient received 8, once-weekly intravesical instillations of 50 mega units of interferon. Response was assessed at formal cystoscopy one to three months from the completion of treatment and a comparison made with previous patterns of recurrence. Three of the 8 patients with papillary tumours had no recurrences, and a further three an apparent reduction in the rate of tumour recurrence with less than 50% of expected tumour present. No patient with flat-in-situ carcinoma responded. Although the response to interferon was less than that with conventional intravesical chemotherapy, these results suggest that further investigation of topical interferon is warranted.

### A comparison of intranasal and depot preparations of buserelin in the management of advanced prostatic cancer.

J.H. Waxman[1,2,3], J. Sandow[4], A. Man[1], M.J. Barnett[1], & R.T.D. Oliver[1,2,3]

[1]*St. Bartholomew's,* [2]*The London Hospital,* [3]*Institute of Urology, London, and* [4]*Hoechst Pharmaceuticals, Switzerland*

Buserelin (D-Ser (TBU) 6 LHRH Ethylamide), a long-acting analogue of gonadotrophin releasing hormone, has been previously applied to prostatic cancer as thrice or five times daily, intranasal, or daily subcutaneous regimens. Elderly patients may have obvious difficulties in complying with such treatment programmes. A monthly depot preparation of Buserelin, which releases the analogue at a mean daily rate of 150 $\mu g$ was used to treat 12 men with advanced prostatic cancer. The hormonal effects of depot Buserelin were compared with that of 200 $\mu g$ of Buserelin given five times daily as an intranasal preparation to 17 patients. Both regimens resulted in suppression of serum testosterone into the castrate range (less than 2.5 nmol $l^{-1}$) at the end of the fourth treatment week (mean serum testosterone intranasal regimen: 2.3 nmol $l^{-1}$, depot regimen: 2.0 nmol $l^{-1}$).

Reimplantation was not followed by any significant increase in serum testosterone concentrations, indicating effective gonadal suppression. (Mean serum testosterone pre = 2.0 nmol $l^{-1}$ and a 4 h post re-implantation = 1.9 nmol $l^{-1}$). It is concluded that monthly depot Buserelin offers an effective alternative to intranasal therapy.

### Metoclopramide (M) and dexamethasone (D) anti-emesis-high dose metoclopramide (HDM) continuous (C) vs. intermittent infusion (I)

P. Warrington, S. Allan, E. Bayliss, D. Farquhar, M. Cornbleet, J. Macpherson, J. Smyth, & R. Leonard

*Department Clinical Oncology, Western General Hospital, Edinburgh, UK.*

HDM is an effective anti-emetic against cis-platinum (P)-induced emesis and its efficacy is increased in combination with D. HDM is usually administered by I which results in drug peaks, troughs and accumulation. Control of emesis has been reported to require a plasma concentration of 85 $\mu g\,ml^{-1}$. We have compared the blood levels achieved by I and C and correlated these with anti-emetic efficacy. The incidence of side effects was also compared. 28 patients receiving P combinations were randomised to receive either IM+D or CM+D and received the alternate regimen on subsequent treatment. IM regimen comprised M 7 mg $kg^{-1}$ in 500 ml 0.9% sodium chloride administered in 100 ml aliquots 15 min before P and thereafter 2 hourly × 4. CM regimen comprised a 3 mg $kg^{-1}$ loading dose of M 15 min before P then continuous infusion of 4 mg $kg^{-1}$ for 8 h. D 20 mg was given by a 15 min infusion 30 min before P. Nausea, retching, vomiting, diarrhoea and adverse reactions were recorded on a standard questionnaire. CM regimen gave a significant reduction in mean nausea (CM = 16% IM = 35% $P<0.001$, visual analogue scale) significant reduction in vomiting ($\geq 3$ episodes CM = 4 IM = 12, $P<0.05$) significant reduction in the incidence of diarrhoea ($P<0.05$).

*Mean plasma levels of metoclopramide in 14 patients ($\mu g\,ml^{-1}$)*

| *Time (h)* | *IM trough* | *IM peak* | *CM* |
|---|---|---|---|
| 0 | — | 0.62±0.25 | 1.47±0.56 |
| 1 | — | — | 0.98±0.16 |
| 2 | 0.21±0.60 | 0.82±0.18 | 0.89±0.18 |
| 4 | 0.44±0.16 | 1.03±0.25 | — |
| 5 | — | — | 0.93±0.31 |
| 6 | 0.52±0.17 | 1.21±0.26 | — |
| 8 | 0.75±0.25 | 1.34±0.34 | 0.99±0.35 |

The mean CM blood levels did not fall below 0.85 $\mu g\,ml^{-1}$ whereas the IM regimen resulted in M levels below 0.85 $\mu g\,ml^{-1}$ during the first 4 h. The CM gives a better anti-emetic control with fewer side effects compared with IM.

### Advanced squamous carcinoma of the lung. Trials of etoposide *vs* no treatment and etoposide *vs* etoposide + cyclophosphamide

G. Anderson[1] & E. Peel[2]

*Lung Cancer Treatment Group. [1]Newport Chest Clinic, Newport, NP9 4GA. and [2]Wallsend Chest Clinic, Tyne and Wear, NE28 7PE, UK.*

There is no evidence that chemotherapy prolongs life in advanced squamous carcinoma of the lung. Most trials have concentrated on the remission incidence with few studies of the effect of treatment on survival.

In two co-operative studies centres entered patients either into randomised trials of no treatment *versus* oral etoposide 300 mg $m^{-2}$ (O *vs* 1) or the same dose of etoposide *vs* the same dose of etoposide + cyclophosphamide 300 mg $m^{-2}$ i.v. (1 *vs* 2). Patients had biopsy proven squamous carcinoma considered too advanced for radical radiotherapy or for surgery. Palliative radiotherapy could be given up to a dose of 3,000 cGy. Patients receiving active treatment received 6 Cycles of chemotherapy.

In the comparison of 0 *vs* 1 for the whole group there was no significant benefit of treatment ($P=0.37$ on log rank analysis median survival on treatment or no treatment 194 days). In 39 patients with $M_1$ disease there was a significant improvement in survival ($P=0.045$ median survival no treatment 103 days, etoposide 251 days). There was no significant benefit of treatment for patients with $M_0$ disease. It is surprising that benefit emerged to the $M_1$ group.

In the study of 1 *vs* 2 there was no survival advantage for the whole group ($P=0.37$ median survival, 1 drug 178 days, 2 drugs 193 days). Separate analysis of $M_0$ and $M_1$ group also showed no significant survival advantage.

### A comparative study of two methods of treating oesophageal cancer related to prognosis

C.T. Doyle[1], M.M. Cole[2] & D.G. Barry[2]

[1]*Department of Pathology and* [2]*Department of Statistics, University College, Cork, Eire.*

One hundred and forty eight patients with squamous cell carcinoma of the oesophagus were retrospectively divided into two groups of 32 men and 42 women. One group had been treated palliatively by intubation, the other surgically by oesophagectomy. Age, sex, duration of symptoms, location of the cancer, differentiation and survival time from diagnosis were coded for both groups, and extent and type of invasion, local tissue reaction, and any associated mucosal premalignant lesions for the oesophagectomy group.

Survival data were analysed using the proportional hazards regression model and the P1L and P2L programmes of the BMDP statistical software package.

The best prognosis was found among women, under 60, treated by oesophagectomy, who had well differentiated cancers in the lower third of the oesophagus, with no premalignant lesions in the adjacent mucosa. Patients with the worst prognosis were also treated by oesophagectomy and had moderately differentiated cancers in the upper and middle thirds. The results allow identification of four prognostic groupings – 'Good', 'Fair', 'Poor' and 'Bad' which in future should assist the decision between palliation and oesophagectomy.

### High-grade non-Hodgkin's lymphomas: Long term results of treatment with combination chemotherapy

D. Cunningham[1], A. Hepplestone[2], N.L. Gilchrist[1], J.H. Dagg[2], I.L. Evans[3] & M. Soukop[1]

[1]*Department of Medical Oncology, Royal Infirmary, Glasgow G4 0SF, Departments of* [2]*Medicine and* [3]*Haematology, Western Infirmary, Glasgow G11 6NT, UK.*

High-grade non-Hodgkin's lymphomas are a heterogenous group of tumours which have a poor prognosis unless treated with chemotherapy. Using regimens such as MOPP and BACOP long term survival has been reported in up to 40% of patients which has led to a reluctance of clinicians to use more intensive chemotherapy regimens which offer the prospect of improved survival, and ultimately cure.

Between 1975 and 1982 in 2 centres in Glasgow, 53 previously untreated patients with high grade non-Hodgkin's lymphomas received chemotherapy consisting of one of the following regimens – CVP, MOPP, CHOP or BACOP. Twenty nine patients (55%) entered complete remission (CR), 20 (38%) had partial remission (PR) and 4 (7%) had progressive disease (PD). Of the 29 patients CR; 9 have relapsed and died, 5 died of infection, 4 died of non-malignant causes and 11 (21%) remain alive and disease free. The median survival for the CR group is 40 months. All of the patients in the PR and PD groups are dead, median survival 11 months.

These chemotherapy regimens will cure only the minority of patients with aggressive lymphomas and the use of more intensive regimens in the management of this disease is indicated.

### Weekly outpatient chemotherapy (EMOP/CA) for non-Hodgkin's lymphoma and relapsed Hodgkin's disease

J.A. Ledermann, D. Pektasides, L. Holden, G.J.S. Rustin & E.S. Newlands

*CRC Department Medical Oncology, Charing Cross Hospital, London W6 8RF, UK.*

We have given an intensive 7 drug combination, weekly, on an out-patient basis for 3 months to 14 previously untreated patients (pts.) with 'high-grade' NHL, 17 pts. with relapsed NHL (12 'high-gd.' 5 'low-gd.') and 6 with relapsed Hodgkin's disease (HD). EMOP/CA comprises etoposide $200\,mg\,m^{-2}$, methotrexate $50\,mg\,m^{-2}$, vincristine $1.4\,mg\,m^{-2}$ (max. 2.0 mg) i.v. alternating each week with cyclophosphamide $400\,mg\,m^{-2}$ and adriamycin 20 mg $m^{-2}$ i.v. Prednisolone 100 mg was given on alternate days, to reduce the steroid toxicity, throughout the 12 weeks of treatment.

In the 'high-gd.' NHL group 7 out of 13 evaluable pts. achieved CR (54%) and 4/13 (31%) PR. 2 early deaths occurred due to infection (WHO perf. gd. 3) 5/13 had additional RT. On follow-up 7/14 (50%) pts. remain without evidence of disease at 5–16 months (median 13 mo.) 1 pt. relapsed at 7 months. In relapsed NHL 'high-gd.' 7 (58%) achieved a further CR of 1–6 mo. (med 5 mo.),

2 PR, 1 NC. Responses were seen in all those with 'low-gd.' histology, 2 CR, 3 PR. In HD 2/6 had a CR and 4/6 PR.

The toxicity has been acceptable with occasional modifications in the dose of steroids and inclusion of folinic acid rescue in those with mucositis. I.v. antibiotics for infection were rarely required. This intensive weekly regimen has produced encouraging results; the schedule is similar to MACOP-B of Klimo and Connors (*Ann. Int. Med.* (1985), **102,** 596) who treated 61 patients but the toxicity is less.

### Cutaneous malignant melanoma tumour type in Northern Ireland: Possible aetiological implications

L.G. Gordon & W.S. Lowry

*The Queen's University, Belfast BT9 7BL, UK.*

The evaluation of all cases of malignant melanoma in Northern Ireland over a 5 year period encompasses a total of 240 cases of cutaneous malignant melanoma (CMM). The distribution of tumour types in this study reveals that 42% are nodular lesions (NM), and 27% are superficial spreading melanoma (SSM); 20% are lentigo malignant melanoma (LMM), and 11% are acral lentiginous melanoma (ALM). This is one of the highest percentages of LMM so far reported.

All tumour types have a majority of lesions greater than 1.5 mm in thickness. Even LMM has 70% of lesions thicker than 1.5 mm. Nodular and ALM lesions are most ulcerated. Anatomical site distribution shows significant differences between types. ALM is most common on the foot (40%). LMM, not surprisingly, is most common on the head and neck (86%). SSM is most common on the leg (43%). NM reveals its highest incidence on the leg (39%) and head and neck (23%). The excess incidence in females in Northern Ireland was evident for all tumour types.

Each of the four types has a statistically different age curve, which together with the variation in site distribution supports a distinctive aetiology for each type. LMM, with highest incidence in the over 65 age groups, follows the age pattern expected with a dose dependent relationship. ALM are most common between the ages of 50 and 70. SSM increases dramatically between the 20–29 age group and the 30–39 group, and then levels out. The age distribution of NM is consistent with the suggestion that many nodular melanomas are actually advanced lesions of SSM and LMM. Studying each tumour type independently helps form a clearer picture of melanoma aetiology from epidemiological evidence.

### Improved curability of teratoma in the West of Scotland – a review of 152 cases

J.D. Graham, S.B. Kayne, D.J. Kerr, L. Mill, & K.C. Calman

*Department of Medical Oncology, Gartnavel General Hospital, Glasgow, UK.*

Between January 1981 and July 1985, 104 patients with testicular teratoma were referred, mainly from urologists in the West of Scotland. Twenty-five had Stage I disease (24%). The remainder received chemotherapy and the volume of metastatic disease has been graded using the system recently proposed by the MRC working party (1985, *Lancet* **i:** 8). By prognostic staging these were as follows: Small volume metastases (SVM) 44 (42%); large volume (LVM) 20 (19%); very large volume (VLVM) 15 (14%). All patients with metastatic disease were treated with platinum containing regimes according to EORTC urology group protocols. A total of 8 patients died: 1/44 with SVM, 0/20 with LVM and 7/15 with VLVM. Thus, 71 out of 79 patients (90%) presenting with metastatic disease are alive, 69 of whom have been off treatment for 3–53 months. During the same period 6 out of 8 patients treated for extragonadal teratoma died.

In contrast 13 out of 35 patients treated for testicular teratoma between 1975 and 1980 died. By Stage these were as follows: Stage I 10 (28%); SVM 10 (28%), 2 dead; LVM 6 (17%), 3 dead; VLVM 9 (25%), 6 dead. There were five patients treated for extragonadal teratoma with 4 deaths.

These data show that an increasing proportion of patients now present with a smaller metastatic load and that virtually all can be cured. The small number of patients presenting with VLVM and extragonadal disease continue to have a poor prognosis indicating a need for alternative chemotherapy schedules for this subgroup.

### Sister chromatid exchange frequency in the lymphocytes of patients with lymphomas having first-line chemotherapy

T. Brown[1], A.A. Dawson[2], B. Bennett[2] & J.L. Watt[1]

[1]*Department of Genetics,* [2]*Department of Medicine, University of Aberdeen, Aberdeen Royal Infirmary, Foresterhill, Aberdeen, AB9 2ZB, UK.*

We have already demonstrated dramatic changes in the sister chromatid exchange (SCE) frequency in the peripheral lymphocytes of patients having MVPP therapy for Hodgkin's disease; the elevation rising rapidly but falling before the end of the course. With CHOP therapy used in treating high-grade non-Hodgkin's lymphoma, a comparatively minor rise was demonstrated in the samples taken immediately before each pulse of therapy.

When samples were analysed in the first day after intravenous therapy however, a pattern of steep rise at 2 h, and fall at 24 h was seen with MVPP therapy, whereas CHOP therapy produced a far greater rise at 2 h, and increasing still further at 24 h.

The findings are of potentially great significance, especially with respect to therapy-induced malignancy.

**A study of the problems that cancer poses for the patient and the family**

M. Casey, R. Conroy, L. Daly, J. Fennelly, P. Herity, N. Hilliard, M. McCambridge & M. Moriarty

*St. Luke's Hospital, Cancer Research Unit, Rathgar, Dublin 6, Eire.*

A study of 200 cancer patients and the problems that cancer poses for them was carried out in St. Luke's and St. Vincent's Hospital between May 1983 and May 1984; one responsible relative for each patient was also included in the study.

The main areas of concern were communication with health professionals, physical and psychological symptoms and their management, social dependency, care in hospital and at home, and financial and social aspects of the illness.

In the four weeks prior to the interview the patients had distress from

| *Symptom* | %+ | *% Gained Relief* | *% No relief or did not seek it* |
|---|---|---|---|
| Lassitude | 65.5 | 16.0 | 84.0 |
| Insomnia | 53.0 | 64.0 | 36.0 |
| Pain | 52.0 | 64.0 | 36.0 |

There was a significant relationship also to anxiety and depression.

**Coping with cancer: A randomised study of relaxation training (ReT) in patient care and management**

S. Bindemann, S.B. Kaye, J. Welsh, T. Habeshaw & K. C. Calman

*Department of Clinical Oncology, University of Glasgow, 1 Horselethill Road, Glasgow G12, UK.*

A randomised trial of Relaxation Training (ReT) was carried out with the purpose of evaluating this form of psychologic support for cancer patients. Seventy one new patient-referals (32 males 39 females) completed the study. Measurement of effect was achieved by means of a 'battery' of questionnaires. These included, the Leeds Self-Assessment of Anxiety (LSA) and of Depression (LSD) Scale, the Stait-Trait Anxiety Inventory (STAI), the General Health Questionnaire (GHQ–60) – a psychiatric disorders screening test and the Actual/Ideal-Self Perception Questionnaire. (Bindemann *et al* 1984, *Br. J. Cancer,* **49:** 387.) A structured interview schedule was developed for use in the study. Baseline data were gathered at recruitment and evaluation of ReT took place at 6 and 12 weeks. Raised anxiety scores of male control Ss – compared to scores reported for male experimental subjects – were noted at both 6 and 12 weeks. ($P = <0.01$) Greater confidence levels emerged in similar differences between female groups ($P = <0.001$). Male group depression scores were statistically comparable throughout the life of the study. However, depression scores for female control Ss were higher at 6 and 12 weeks ($P = <0.01$ and $P = <0.001$). Elevated GHQ scores were reported for male control Ss at 12 weeks only ($P = <0.05$). Similar differences, but in this instance at both 6 and 12 weeks, distinguished the two female groups ($P = <0.05$). Groups' placement on the factor of intrapsychic functioning ascribes a lower level of psychic well-being to male control Ss at 6 and 12 weeks ($P = <0.05$). Differences on this variable were again most apparent between female control Ss (lower) and female experimental Ss (higher) at 6 and at 12 weeks ($P = <0.02$, $P = <0.002$). Quality of life, as measured by specific references to actual- and ideal-self perception, was more adversely affected among control subjects of both sexes. Data obtained by clinical interview, undertaken by members of medical staff within the Department, concur fully with results summarized above. In conclusion, we suggest that these results ascribe value to ReT as a useful means of supporting cancer patients, particularly female patients, in their need to mobilize and maximize upon actual coping resources.

**Familial medullary thyroid cancer – screening the family of the apparently sporadic case**

B.A.J. Ponder
(for the CRC Medullary Thyroid Group)

*Institute of Cancer Research, Sutton, Surrey, SM2 5PX, UK.*

Medullary Thyroid Cancer (MTC) occurs in sporadic and familial (autosomal dominant) form. About 10% of new cases of MTC are recognisable as familial on history: the other 90% are categorised as 'apparently sporadic'.

The value of early diagnosis by calcitonin measurement in families at risk is well established. Few families of patients with apparently sporadic MTC are offered screening, however, probably because it is believed that onset in middle age or later and lack of immediate family history exclude the familial form with high probability.

Analysis of data from 42 known MTC families and 37 consecutively screened 'apparently sporadic' families in the CRC group register showed (1) that a minimum of 15% of consecutive unselected 'sporadic' families screened were in fact familial, (2) that of a total 12 'sporadic' cases found by screening to be familial 6 were initially diagnosed aged >40 years, and (3) that 30% of obligatory gene carriers in known families will still not be diagnosed clinically by age 70. Potentially, 2/3 of new families with MTC will be discovered by screening, and only 1/3 by family history. Advanced age at diagnosis in the index case, and lack of history even in elderly parents, are not sufficient to rule out familial involvement.

We conclude that screening should be considered for the family of every new case of MTC. Categorisation of 'apparently sporadic' families as high- or low-risk may in future be possible using the family structure combined with age at onset data derived from the register.

### Augmentation of human NK cell activity by cloned NS1 influenza viral gene products

R.C. Rees[1], B.J. Dalton[2], & J.F. Young[3]

[1]*Department of Virology, University of Sheffield Medical School, UK,* [2]*Departments of Immunology and Anti-infectives Therapy and* [3]*Molecular Genetics, Smith, Kline and French Laboratories, Philadelphia, USA.*

*E. coli* cloned influenza viral gene products were assessed for their ability to augment human natural cytotoxicity in overnight cultures (18 h) at 37°C. Nylon wool non-adherent PBMC were activated by a number of viral gene proteins, the most effective being the NS1 protein (but not NS2 protein) and haemagglutinin and matrix antigen components fused to the N-terminal 81 amino acid sequence of NS1. Interferon (IFN) was detected in cultures where enhanced cytotoxicity was evident and identified as both IFN$\alpha$ (>90%) and IFN$\gamma$ (<10%). The cell type responsive to antigen stimulation was present in Percol fractions enriched for LGLs, and NS1 activated PBMC were shown to localise in the low density Percol fractions (LGL enriched). Using specific anti-IFN-antisera it was determined that IFN$\alpha$, but not IFN$\gamma$, was responsible for enhancing cytotoxicity. Interferon induction and activation of cytotoxicity could not be ascribed to the presence of contaminated bacterial products. These studies suggest that NS1 protein and constructs containing a portion of the NSI antigen augment human NK cells via the induction of IFN.

### Tissue-type plasminogen activator: correlation with oestradiol receptors in human breast carcinomas

M.J. Duffy, P. O'Grady & H.R. Lijnen

*St. Vincent's Hospital, Dublin, Eire and University of Louvain, Belgium.*

Plasminogen activator (PA) is a protease which catalyses the conversion of the inactive plasminogen to the active plasmin. In most tissue it exists as 2 main forms, i.e. tissue-type PA (t-PA) and urokinase-type PA (UK-PA). Since PA is induced by oestradiol in both rat uteri and human breast cancer cells in culture, its presence in human breast cancer biopies is a potential marker for a functional oestradiol receptor (OER). The purpose of this investigation was therefore to see if any correlation existed between OER and either total PA, or its different forms in human breast tumours.

t-PA as measured by an immunoradiometric assay was found in 1/31 (3.2%) of tumours without OER, in 4/13 (31%) of tumours with borderline level of OER and in 49/94 (52%) of tumours with OER ($P<0.001$ for ER-negative versus ER-positive group). In contrast to t-PA, neither UK-PA activity nor total PA activity showed any significant relationship with OER. Other oestrogen-inducible proteins such as peroxidase and creatine kinase also showed no significant correlation with OER.

Our results show that t-PA antigen is mostly confined to OER-containing carcinomas. Tumours possessing OER and t-PA may contain a functional receptor while those containing OER but lacking t-PA may have an inactive receptor. If so OER-positive, t-PA positive tumours would be expected to respond to hormonal therapy while OER-positive, t-PA-negative carcinomas would not. This hypothesis is currently being tested.

**Increased levels of tissue plasminogen activator mRNA during progressive transformation of ethylnitrosourea-induced rat brain cells**

L.J. Green, P.C. Rumsby and J.P. Roscoe

*Department of Cell Pathology, School of Pathology, Middlesex Hospital Medical School, Riding House Street, London W1P 7LD, UK.*

A system for investigating changes in the progressive transformation of rat brain cells has been developed using ethylnitrosourea (ENU) as the inducing carcinogen (Roscoe & Claisse, (1976), *Nature,* **262,** 314). A series of cultures derived at different times after *in vivo* exposure to this carcinogen as well as cultures from tumours and control animals has allowed the identification of stages during transformation. Differences in plasminogen activator (PA) activity have proved to be of particular interest in this transformation system (Roscoe *et al.,* (1980), *Br. J. Cancer,* **42,** 756).

Using a fibrin-agarose overlay assay a higher level of PA activity has been found in the tumour cultures than in control cultures derived from adult rat brain. Northern blot analysis of mRNA demonstrated a higher level of tissue-type plasminogen activator (t-PA) mRNA in rat glioma than in normal rat brain cultures. Cultures derived 2 days after exposure to ENU had low PA activity at early passages. On further passaging, there was an increase in PA activity which preceded the ability of cells to grow in soft agar or syngeneic animals. The tPA mRNA level in these cells was also low initially and increased on passaging, correlating with the rise in enzyme activity found during the progression of the cells to the fully transformed phenotype. Control cultures (buffer exposed) had no measurable PA activity of tPA mRNA even after comparable passaging. The results open the way to investigations at a molecular level of alterations in the control of a specific proteolytic enzyme early in transformation.

**Glycoprotein expression in normal keratinocytes and squamous carcinoma cell lines**

Z. Rayter[1] & R.A.J. McIlhinney[2]

[1]*Ludwig Institute for Cancer Research, The Haddow Laboratories, Royal Marsden Hospital, Clifton Avenue, Belmont, Sutton, Surrey, SM2 5PX,* [2]*MRC Unit of Anatomical Neuropharmacology, University Department of Pharmacology, South Parks Road, Oxford, UK.*

Glycoprotein expression in normal keratinocytes has been compared with that in the squamous carcinoma cell lines LICR-HN2, -HN5, -HN6 and on SV40 transformed keratinocyte cell line. Two-dimensional gel electrophoresis has been employed to separate detergent-extracted glycoproteins which have then been identified using a $^{125}$I-Con A overlay. A consistent 3 to 4-fold increase in expression of a 35 Kd protein with an iso-electric point of 5 has been observed in the squamous carcinoma cell lines compared to normal keratinocytes. The expression of this protein is decreased in quiescent cells compared with exponentially growing cells. This protein has a similar molecular weight and iso-electric point to that of cyclin or proliferating cell nuclear antigen (PCNA). However, it is affected by mild trypsinization and therefore appears to be on the cell surface.

Cyclin was immunoprecipitated from HN-6 cells labelled with $^{35}$S methionine, and overlay with $^{125}$I-Con A on a two-dimensional gel failed to show any binding. Our conclusion is that we have identified a glycoprotein whose expression is increased in all the squamous carcinoma cell lines, and is not cyclin, the expression of which may be related to cell growth.

**The effect of prolonged tamoxifen treatment on expression of oestrogen receptor by ZR-75-1 human breast cancer cells**

H.W. Van den Berg[1], M. Lynch[1], R. Clarke[2] & J. Nelson[2]

*Departments of* [1]*Therapeutics and Pharmacology and* [2]*Biochemistry, Queen's University of Belfast, UK.*

Continuous tamoxifen, (Tam), therapy is of proven value in prolonging disease free interval in breast cancer patients. The effects of such treatment on surviving tumour cells, or the consequences of cessation of therapy are largely unknown. We have maintained ZR-75-1 human breast cells in growth medium containing tamoxifen, (1–2 $\mu$M), for 6 months. Cells, designated ZR-TAM, grow slowly in the presence of the antioestrogen, requiring subculturing every 2–3 weeks. Determination of oestrogen receptor, (ER), content 5 days after transfer to drug free medium was achieved using a whole cell binding assay. Using free $^{3}$H-oestradiol concns. ranging 0.3 to 3.6 nM a single class of ER receptor was detectable in ZR-75-1 cells, (Bmax 225 $\pm$ 19 (s.e.) fmol mg$^{-1}$ protein, Kd 0.57 $\pm$ 0.11 nM). Expression of this high affinity receptor was markedly reduced in ZR-TAM cells, (Bmax 56 $\pm$

12 fmol $mg^{-1}$ protein, Kd 0.21 ± 0.08 nM). Woolf or Scatchard analysis of binding data also revealed the presence of low affinity binding sites in ZR-TAM cells. Preliminary data have demonstrated saturability of these sites at high free ligand concentrations with binding characteristics similar to those of previously described nuclear type 2 sites, (Kd ≃ 10–8M). In contrast to the results of earlier experiments, ZR-TAM cells grew at the same rate as ZR-75-1 cells in absence of Tam and cell proliferation was inhibited to the same extent on re-exposure to Tam. We conclude that human breast cancer cells can withstand prolonged exposure to Tam without resistance occurring, although the expression of high affinity ER is greatly reduced. We are currently investigating the possible role of the low affinity ER binding sites in mediating the effects of Tam.

### Cytotoxic drugs induce a reduction in the 17β oestradiol ($E_2$) binding capacity of MCF-7 human breast cancer cells which is accompanied by a reduction in the rate of DNA synthesis

R. Clarke[1], J. Morwood[1], J. Nelson[1], H.W. van den Berg[2] and R.F. Murphy[1]

*Departments of [1]Biochemistry and [2]Therapeutics, The Queen's University of Belfast, N. Ireland, UK.*

MCF-7 cells were exposed to clinically achievable concentrations of adriamycin (ADR), melphalan (MEL) and 5-fluorouracil (5-FU) for 24 h prior to estimating the oestrogen receptor (ER) levels using a whole cell binding assay.

ADR, MEL and 5-FU reduced the ER content of MCF-7 cells in a dose dependent manner, but the affinity of remaining ER for the ligand, the uptake of the ligand by the cells, and the rates of protein synthesis and cell proliferation were not significantly different from untreated cells. A reduction in the $E_2$ binding capacity of the cell population was accompaneid by a reduction in the rate of newly synthesised DNA.

These results suggest that the reduction in ER levels is not the result of reduced synthesis of new ER protein. The ER is currently thought to be predominately located in the nucleus where it becomes more tightly associated on binding $E_2$ (Molinari *et al*, (1985), *Biochem. Biophys. Res. Comm.*, **128,** 634). Previously bound $E_2$ may not be available to exchange with radiolabelled $E_2$. Therefore, the only ER accessible for binding may be that associated with newly synthesised DNA. Alternatively, the cytotoxic drugs may be influencing the rate of ER recycling.

These observation may have important implications for ER determination in patients previously treated with cytotoxic drugs as well as for combined regimes using cytotoxic drugs and antioestrogen therapy.

### Conditioning factors affecting growth of human skin keratinocytes

S. McDonnell and M. Clynes

*School of Biological Sciences, National Institute for Higher Education, Glasnevin, Dublin 9, Eire.*

Irradiated (or mitomycin C-treated) 3T3 feeder layers are generally required for primary culture of disaggregated human skin keratinocytes (HSK), even when subsequent subcultures can be achieved without feeders (Peehl & Ham, (1980), *In Vitro,* **16,** 516). In serum-free optimized media normal HSK show density-dependent effects (Tsao *et al.,* (1982), *J. Cell Physiol,* **110,** 219) and we have observed a low-density cut-off point in SCC-9, a line derived from squamous cell carcinoma of the tongue (Rheinwald & Beckett, (1982), *Cancer Res.,* **41,** 1657). These 3T3 and SCC-9 feeder effects are mediated at least in part by diffusible factors; we have found, using SCC-9 as an indicator line, that both 3T3 and SCC-9 can act as feeders in a double-layer agar assay, and that conditioned media (CM) from both lines increases plating efficiency and colony size in monolayer assay. SCC-9 CM is also active in the transforming growth factor (TGF) assay, using NRK as the indicator line, and this activity is associated with material of >5,000 mol. wt. The 3T3 and SCC-9 products described here may be useful in improving growth conditions for normal and malignant HSK, and may possibly have relevance to *in vivo* control of epithelial cell proliferation.

### *In vivo* susceptibility of dormant carcinoma cells to alkylating agents

P.V. Senior & P. Alexander

*CRC Medical Oncology Unit, Southampton General Hospital, Southampton SO9 4XY, UK.*

We have established a transplantable mammary carcinoma syngeneic in the hooded Lister rat which will only grow in rats bearing a subcutaneously implanted pellet of oestrogen. In the absence of exogenous oestrogen transplanted carcinoma cells remain dormant, but when the rats receive an oestrogen pellet growth commences at a rate comparable to that of transplants made into rats already receiving oestrogen. Carcinoma cells were either inoculated s.c., when they gave rise to locally growing tumours, or into the arterial circulation, via a cannula introduced into the left ventricle, when metastases grew predominantly in the adrenals, ovaries, bone and lung. The response of dormant and actively dividing carcinoma cells to alkylating agents was compared by administering the alkylating agents either before or after implantation of the oestrogen pellet. Cyclophosphamide in the dose range of 40–180 mg $kg^{-1}$ given during the dormant phase caused no delay in growth which occurs after oestrogen had been given for the s.c. tumour and only produced a marginal effect on the metastases. Cyclophosphamide was, however, highly effective in delaying both local and metastatic tumour growth when given after oestrogenisation (i.e. when the carcinoma cells were actively growing). On the other hand, BCNU at 7 mg $kg^{-1}$ was highly effective against this carcinoma, both in the the dormant state and in the actively growing phase. These findings may be relevant to the design of protocols of adjuvant chemotherapy since dormancy may contribute to the resistance of some micro-metastases.

**Characterisation of vesicles shed from Landschutz ascites tumour in association with malignancy-related fucopeptides**

M. McGuinness[1], E. Walsh[2] & H. Smyth[1]

[1]*Department of Biochemistry, University College, Dublin 4,* [2]*School of Biol. Sciences, N.I.H.E., Dublin 9, Eire.*

The membrane glycoproteins of malignant cells are abnormally enriched in large highly sialylated fucopeptides and incubation of cells with trypsin is widely used to release these entities. Using Landschutz ascites tumour we previously showed that these large fucopeptides are released in association with the vesicle fraction of cell-free supernatants. Some release occurs spontaneously into saline (PBS). This is increased in the presence of trypsin in association with increased vesiculation. Fucopeptide size profiles are similar for vesicles shed under these two conditions. The present study concerned further examination of these vesicle fractions released from cells incubated in PBS alone or containing trypsin (0.1 mg $ml^{-1}$). Metabolic radioactive labelling showed trypsinate vesicles to contain more cholesterol ($\times 2.5$), phospholipid ($\times 5$) and fucose ($\times 1.7$) than control vesicles from the same number of cells. Cholesterol and phospholipid were also determined chemically. Spontaneous release of the malignancy-related fucopeptides was found to be associated with a vesicle fraction having a cholesterol/phospholipid mole ratio double that of the bulk plasma membrane, i.e. representing more rigid domains. In contrast, trypsinate vesicles gave a ratio similar to that of the plasma membrane. The association of these glycopeptides with vesicles indicates that the parent glycoproteins are integral membrane proteins and that they are only indirectly sensitive to trypsin.

**Fluorescent microscopic studies on the differential cellular distribution of adriamycin and 4′-deoxydoxorubicin.**

J.A. Plumb, N. Wilmott, D.J. Kerr, A.D. Burt, I.A.R. More & S.B. Kaye

*Department of Medical Oncology, University of Glasgow, Glasgow G12 9LX, UK.*

The multicellular spheroid was developed as a system of intermediate complexity between solid tumours and cell monolayers. Penetration barriers have been postulated for adriamycin on the basis of fluorescent microscopic and flow cytometric studies on spheroids of V79 Chinese Hamster ovary cells. We have compared the differential distribution of adriamycin and 4′-deoxy-doxorubicin (4′-deoxy) a lipophilic derivative, by fluorescent microscopy in monolayers and spheroids of a human non-small cell lung tumour and in a solid tumour grown subcutaneously in rats. At identical drug concentrations and duration of exposure, adriamycin bound to cell nuclei whereas 4′-deoxy was distributed predominantly in a granular fashion in the cytoplasm with some nuclear binding in cell monolayers. Ultrastructural studies suggest that 4′-deoxy might be binding to cytoplasmic lysosomes. In spheroids from the same cell line adriamycin (5 $\mu g\,ml^{-1}$ for 2 h) was seen in the nuclei of the outer 3–4 cell line layers whereas 4′-deoxy (same concentration) had penetrated further to a depth of 6–7 cell layers. The drugs were infused (80 mg $kg^{-1}$ over 1 h) via the carotid artery of SP107 adenocarcinoma bearing male rats. Fluorescent microscopy of frozen sections showed a faint ring of fluorescence on the periphery of the tumour which corresponded to the position of the majority of blood vessels (demonstrated by a fluorescent antibody directed against factor VIII). 4′-deoxy penetrated further from the outer vascular ring than did adriamycin. We believe that these data support the hypothesis that drug penetration barriers exist for adriamycin and that these may be overcome, partially by the lipophilic analogue.

**Inhibitors of ADP-ribosyl transferase protect against the cytotoxicity of S-phase acting drugs**

K. Moses, A.L. Harris & B.W. Durkacz

*Cancer Research Unit, Royal Victoria Infirmary, Newcastle upon Tyne NE1 4LP, UK.*

The synthesis of poly(ADP-ribose) by ADP-ribosyl transferase (ADPRT) is inhibited by a number of benzamides. These ADPRT inhibitors enhance the cytotoxicity of DNA damaging agents and retard DNA excision repair, without themselves affecting cell viability. At higher inhibitor concentrations, cells arrest in G1 or G2. We investigated the effects of 3 ADPRT inhibitors (3-aminobenzamide, 6-methoxybenzamide and 3-acetamidobenzamide) on 2 S-phase acting drugs, hydroxyurea (HU), and 5 fluoro-2′-deoxyuridine (FUdr). Log-phase CHOKI cells were preincubated for 8 h with inhibitors, exposed to HU or FUdr for 16 h, and plated for survivors without drugs. All 3 inhibitors reduced the cytotoxicity of HU and FUdr in a dose dependent manner. For example, 2 mM HU reduced survival to 1%, but with 20 mM 3-aminobenzamide, survival increased to 50%. A significant increase in survival was observed with 1 mM 3-aminobenzamide, a concentration 5 × lower than that used to inhibit DNA excision repair. We interpret these results as follows: inhibition of ADPRT results in a reversible cell cycle block in G1 or G2. Cells are prevented from entering S-phase during exposure to HU or FUdr, thus alleviating the cytotoxic effects. Of particular significance is the observation that cell cycle blocks are detected at concentrations of inhibitors reported not to affect cell proliferation.

**Modulation of transforming growth factor production in chemically transformed rat glioma cells by passaging and mitomycin C**

A.M. Owsianka, R. Zammit-Pace and J.P. Roscoe

*Department of Cell Pathology, School of Pathology, Middlesex Hospital Medical School, London W1P 7LD, UK.*

Tumourigenic cells obtained after induction in rats by the neurotropic carcinogen, ethylnitrosourea (Roscoe (1980) *Br. Med. Bull.*, **36**, 33), were examined for production of transforming growth factor (TGF) activity. The cells were plated in petri dishes and TGF activity detected by the ability to induce colony formation in an overlay of rat kidney cells (NRK 49F) in agar. Several lines produced significant TGF activity. Surprisingly, the results showed that some lines produced more TGF activity at earlier passages than at later passages when the cells themselves have a higher plating efficiency in agar. It was also observed that these later passage cells grew more extensively under the NRK cells. Treatment with Mitomycin C prior to overlaying with NRK cells resulted in an enhancement of TGF production. Small scale partial purification of conditioned medium from low passages of a good producer line was carried out. The material obtained had TGF activity which was not potentiated by adding epidermal growth factor. Relative to its ability to induce NRK growth it was a poor competitor for EGF receptor binding. Purification of conditioned medium from high passage cells showed that this had much less TGF activity which in contrast was potentiated by EGF. The results showed that TGF production is altered by passaging and growth inhibition. Increased plating efficiency of these glioma cells in agar need not be associated with higher TGF secretion. However, the cells could still be producing and responding to their own TGF.